# CircPCNXL2 promotes tumor growth and metastasis by interacting with STRAP to regulate ERK signaling in intrahepatic cholangiocarcinoma

**DOI:** 10.1186/s12943-024-01950-y

**Published:** 2024-02-17

**Authors:** Shuochen Liu, Yirui Wang, Tianlin Wang, Kuangheng Shi, Shilong Fan, Chang Li, Ruixiang Chen, Jifei Wang, Wangjie Jiang, Yaodong Zhang, Yananlan Chen, Xiao Xu, Yue Yu, Changxian Li, Xiangcheng Li

**Affiliations:** 1 Hepatobiliary Center, Key Laboratory of Liver Transplantation, The First Affiliated Hospital of Nanjing Medical University, Chinese Academy of Medical Sciences, NHC Key Laboratory of Living Donor Liver Transplantation (Nanjing Medical University), Nanjing, Jiangsu Province China; 2 Wuxi People’s Hospital, Wuxi Medical Center, The Affiliated Wuxi People’s Hospital of Nanjing Medical University, Nanjing Medical University, Wuxi, Jiangsu Province China

**Keywords:** Intrahepatic cholangiocarcinoma, CircPCNXL2, STRAP, MEK/ERK signaling, Trametinib

## Abstract

**Background:**

circular RNAs (circRNAs) have been reported to exert important effects in the progression of numerous cancers. However, the functions of circRNAs in intrahepatic cholangiocarcinoma (ICC) are still unclear.

**Methods:**

circPCNXL2 (has_circ_0016956) were identified in paired ICC by circRNA microarray. Then, we assessed the biological functions of circPCNXL2 by CCK8, EdU, clone formation, transwell, wound healing assays, and xenograft models. RNA pull-down, mass spectrometry, and RNA immunoprecipitation (RIP) were applied to explore the interaction between cirrcPCNXL2 and serine-threonine kinase receptor-associated protein (STRAP). RNA pull-down, RIP and luciferase reporter assays were used to investigate the sponge functions of circPCNXL2. In the end, we explore the effects of circPCNXL2 and trametinib (a MEK1/2 inhibitor) in vivo.

**Results:**

circPCNXL2 was upregulated in ICC tissues and cell lines, which promoted the proliferation and metastasis of ICC in vitro and in vivo. In terms of the mechanisms, circPCNXL2 could directly bind to STRAP and induce the interaction between STRAP and MEK1/2, resulting in the tumor promotion in ICC by activation of ERK/MAPK pathways. Besides, circPCNXL2 could regulate the expression of SRSF1 by sponging miR-766-3p and subsequently facilitated the growth of ICC. Finally, circPCNXL2 could partially inhibit the anti-tumor activity of trametinib in vivo.

**Conclusion:**

circPCNXL2 played a crucial role in the progression of ICC by interacting with STRAP to activate the ERK signaling pathway, as well as by modulating the miR-766-3p/SRSF1 axis. These findings suggest that circPCNXL2 may be a promising biomarker and therapeutic target for ICC.

**Supplementary Information:**

The online version contains supplementary material available at 10.1186/s12943-024-01950-y.

## Introduction

Intrahepatic cholangiocarcinoma (ICC) is the second most common primary liver cancer, with its incidence and mortality continuing to rise in recent decades [1]. ICC is a highly lethal disease and most patients lose the chance to receive radical surgery when they have symptoms [2]. For patients with unresectable ICC, chemotherapy, radiation therapy, and immunotherapy are of limited effectiveness [3]. The clear mechanisms of ICC have not been fully explained, so exploring further molecular mechanisms of ICC is essential to improve ICC patients’ survival.

Non-coding RNAs (ncRNAs), mainly containing microRNAs (miRNAs), circular RNAs (circRNAs) and long non-coding RNAs (lncRNAs), are regarded as potential biomarkers and therapeutic targets in human diseases including cancers [4]. Circular RNAs (circRNAs) are a kind of non-coding RNA without 5’ N7-methylguanosine caps and 3’ polyadenylated tail, which are mostly generated from the back-splicing of exons of precursor mRNAs, and this unique structure lets circRNAs more stable [5]. Recent studies demonstrated that circRNAs could act as a miRNA sponge, encode a peptide, regulate splicing, and interact with protein during tumorigenesis [6–9]. Currently, several studies demonstrated that circRNAs with abnormal expression may be potential diagnostic and therapeutic targets in many cancers, including ICC, which need to be further studied [10]. To date, there has been limited research on the functions of circRNAs in ICC and the detailed mechanisms of circRNAs in ICC remain to be elaborated.

The Mitogen-activated protein kinase (MAPK) pathways are frequently altered in various diseases, and they play a crucial role in modulating tumor progression and drug resistance in cancers [11]. MAPK pathways are categorized into three subgroups: the extracellular signaling-regulated kinase (ERK) pathway, Jun N-terminal kinases (JNK), and p38 MAPK pathways [12]. The ERK pathway includes RAS, RAF, MEK, and ERK, which altered in almost 40% of cancers, including ICC. Currently, the RAF/MEK/ERK pathway stands out as a promising and clinical significance target and several RAF and MEK inhibitors are in the stage of preclinical or clinical evolution, such as the combination of trametinib with dabrafenib is approved by the FDA for advanced melanoma [13]. However, the relationship between the ERK pathway and circRNAs is still unclear and requires further investigation.

Serine-threonine kinase receptor-associated protein (STRAP), a member of the family of WD-40 repeat proteins, is reported as an oncogene in many cancers, including lung cancer [14], intestinal cancer [15], and hepatocellular carcinoma [16, 17]. STRAP regulates many pathways in human cancers, such as the Wnt/β-catenin pathway, MEK/ERK pathway, and TGF-β signaling [14, 15, 18]. Since there is no study about the functions of STRAP in ICC, it is essential to explore its role in the initiation of ICC formation.

In our present study, we identified a circRNA named circPCNXL2, which is upregulated in ICC based on circRNA sequencing and qRT-PCR analysis. Then, we confirmed that circPCNXL2 could promote proliferation and metastasis in ICC, both in vitro and in vivo. Furthermore, our results revealed that circPCNXL2 could interact with STRAP, enhancing the correlation between STRAP and MEK1/2, which is required in activating ERK signaling. Moreover, we demonstrated that circPCNXL2 acted as a sponge of miR-766-3p, modulating SRSF1 and thereby promoting ERK phosphorylation activity. Finally, circPCNXL2 overexpression could attenuate the tumor-suppressive activity of trametinib (a MEK inhibitor) in ICC. These findings indicate that circPCNXL2 shows promise as a potential target in the therapy of ICC.

## Materials and methods

### Human tissue samples

The tissue was from 76 ICC patients who underwent surgery from 2011 to 2015 at The First Affiliated Hospital of Nanjing Medicine University (No. 2019-SR-133). Patient follow-up continued until death or October 25, 2019. Our research was conducted with the approval of the Ethics Committee of The Affiliated Hospital of Nanjing Medical University. Informed consent was obtained from the patients in accordance with regional regulations.

### RNA-seq and data analysis

The circRNA sequencing data were retrieved from Gene Expression Omnibus (GEO) database (https://www.ncbi.nlm.nih.gov/geo) (GSE181523 [19]). The RNA-seq between circPCNXL2 overexpression and control groups is conducted by Biomarker (China). Total RNA extraction was conducted by TRlzol Reagent (Life technologies, USA). Then, we utilized the Ultima Dual-mode mRNA Library Prep Kit for Illumina (Yeasen Biotechnology, China) to generate sequencing libraries. Library sequencing was performed on Illumina NovaSeq6000 platforms (Illumina, USA). Reads containing adapters, poly-N, and low-quality reads in the raw data were removed. The FASTQ reads were aligned to the GRCh38 reference human genome. Differential expression analysis was carried out by DEseq2 R package in R 4.2.1 with R Studio [20].

### Animal experiment

Four-week-old male BALB/c nude mice were purchased from the Animal Center of Nanjing Medical University. All animal studies were approved by the Nanjing Medical University Ethics Committee (No. 2,307,061). For the xenograft tumor growth assay, 100 µl cell suspensions containing 5 × 10^6^ stable cells (HuCCT1 or RBE) were subcutaneously injected into the axilla of mice. Four weeks after injection, the mice were sacrificed. We measured tumor size every week. The tumor volumes were calculated by the formula (width^2^ × length) / 2, and tumors were weighed. For the liver metastasis model, 5 × 10^6^ stable cells (HuCCT1 or RBE) resuspended into 100 µl PBS were injected into the mouse spleen parenchyma, and subsequently removed to avoid intrasplenic growth. After 4 weeks injection, the mice were sacrificed, and liver tissues were harvested for hematoxylin-eosin (HE) staining.

### Statistical analysis

The statistical analysis was carried out using SPSS 27.0 and GraphPad Prism 9.0 software. Data are shown as mean ± SD. For continuous variables, t test was used for two comparisons. The relationships between circPCNXL2, miR-766-3p, SRSF1 expression and clinicopathological features of ICC patients were calculated by χ2 test, while the correlation between circPCNXL2 and miR-766-3p or SRSF1 expression was analyzed by Pearson’s correlation test. For non-normally distributed continuous data, we employed Wilcoxon rank-sum test. A Cox regression model was employed for multivariate analysis. Survival data were measured by the Kaplan-Meier method and analyzed by the Log-rank test. Differences were considered statistically significant when *p < 0.05, **p < 0.01, or ***p < 0.001.

Additional materials and methods are provided in the Supplementary materials and methods.

## Result

### Identification of circPCNXL2 and circPCNXL2 is upregulated in ICC

**Fig. 1:**
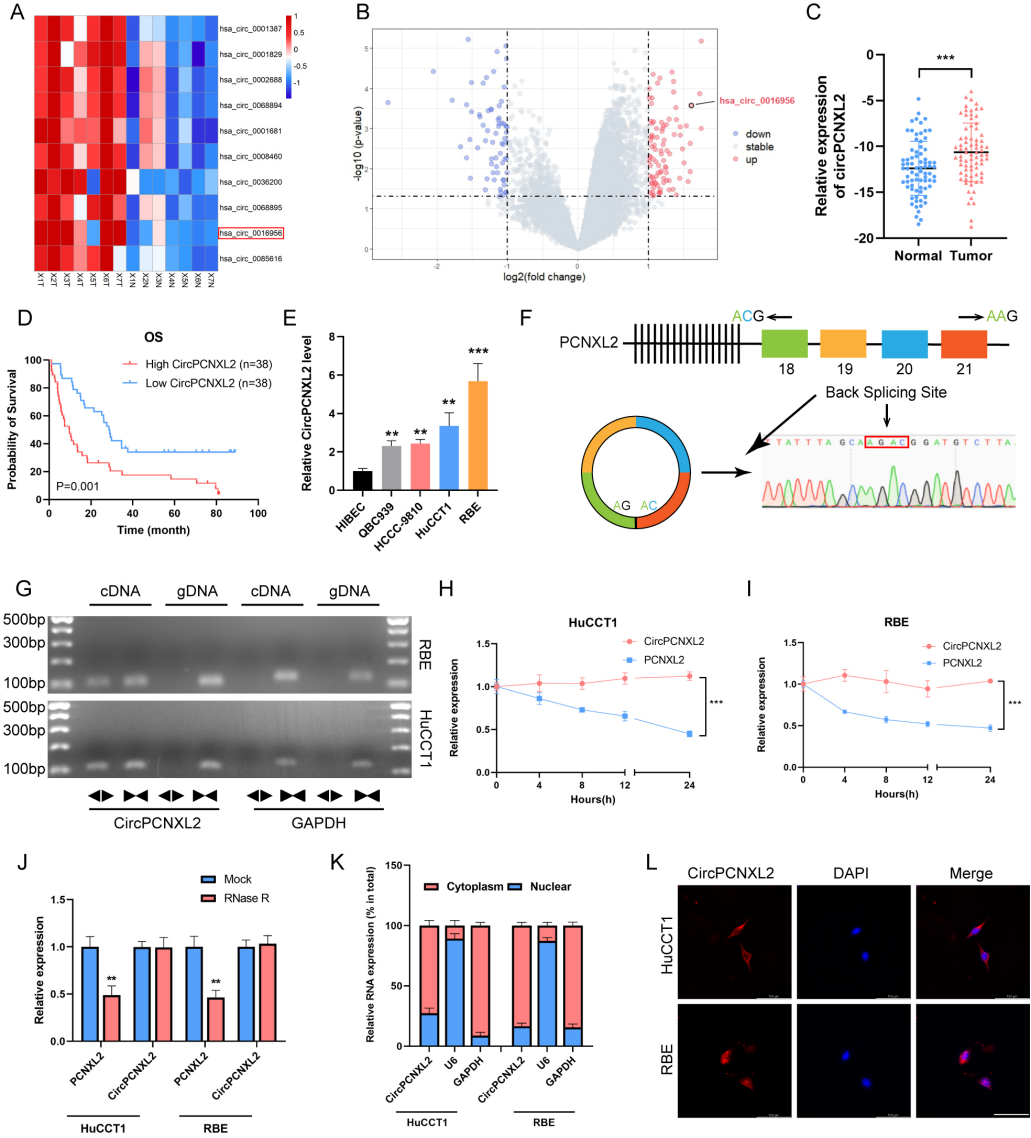
Identification of circPCNXL2 and circPCNXL2 is upregulated in ICC. **(a)** The volcano plot of differentially expressed circRNAs. (**b)** Heatmap of the differentially expressed circRNAs in seven ICC tissues and paired normal tissues by circRNA sequencing. **(c)** Relative mRNA levels of circRCNXL2 in 76 paired ICC and normal tissues by qRT-PCR. **(d)** The Kaplan-Meier overall survival curve of circPCNXL2 in 76 ICC patients. Patients were stratified by the median expression of circPCNXL2. **(e)** Relative mRNA levels of circPCNXL2 in BEC, QBC939, HCCC-9810, HuCCT1 and RBE cell lines. **(f)** Schematic illustration of the genomic location and back splicing of circPCNXL2. Specific divergent primers were designed targeting the back-splice junction and qRT-PCR products were validated by Sanger sequencing. **(g)** The circular characteristic of circPCNXL2 was detected by qRT-PCR and agarose gel electrophoresis using divergent and convergent primers in cDNA and gDNA. **(h-j)** The stability of circPCXNL2 and PCNXL2 was evaluated after Actinomycin D or RNase treatment. **(k)** Nuclear-cytoplasmic fractionation assay identified the localization of circPCNXL2. **(l)** The subcellular location of circPCNXL2 in ICC cells was investigated by FISH assay, scale bar = 100 μm. *P < 0.05, **P < 0.01, ***P < 0.001

To assess the role of circRNAs in ICC, we analyzed the data of RNA-seq from 7 ICC and adjacent tissues [19]. We found 89 circRNAs were upregulated and 67 circRNAs were downregulated in ICC (*p* < 0.05 and log |FC|>1). As shown in the results, has_circ_0016956 (circPCNXL2) is one of the most upregulated circRNAs and its overexpression was confirmed in our 76 paired ICC tissues by qRT-PCR (Fig. 1a-c and Fig. S1a). We categorized the 76 ICC patients into a low circPCNXL2 group and a high circPCNXL2 group based on the median expression of circPCNXL2. We observed that high circPCNXL2 expression was associated with the advanced TNM stage (Table 1). Consistently, patients in the high circPCNXL2 group exhibited poor overall survival (OS) (Fig. 1d). The result of univariate and multivariate analyses identified a high level of circPCNXL2 as a risk factor for OS, together with advanced T stage, positive lymph node metastasis (Table S1). The diagnostic value of circPCNXL2 in ICC was confirmed using the AUC of the ROC curve (Figure S1b, AUC = 0.67, *p* < 0.0001). In conclusion, we proposed that circPCNXL2 had a potential role in the development and metastasis in ICC.

**Table 1:** Relationship between circPCNXL2, miR-766-3p, SRSF1 and clinicopathological characteristics in ICC patients

	circPCNXL2 expression				
Characteristics	High(*N* = 38)		Low(*N* = 38)	p value	
Gender				0.639	
Male	24		22		
Female	14		16		
Age				0.247	
< 60	19		24		
≥ 60	19		14		
T stage				0.791	
T1	29		28		
T2 - T4	9		10		
N stage				0.128	
N0	29		34		
N1	9		4		
TNM stage				0.044*	
I-II	27		34		
III	11		4		
HBsAg				0.479	
Positive	16		13		
Negative	22		25		
Microvascular invasion				0.387	
Yes	9		6		
No	29		32		

The expression of circPCNXL2 is also higher in 4 ICC cell lines, and we selected HuCCT1 and RBE cells for subsequent studies (Fig. 1e). To confirm the circular form of circPCNXL2, Sanger sequencing of qRT-PCR products with divergent primers was employed to confirm its head-to-tail back splicing (Fig. 1f). Divergent primers were designed to amplify circPCNXL2, while convergent primers were used for linear PCNXL2 amplification. Then, we only found circPCNXL2 was amplified from cDNA by divergent primers (Fig. 1g). After treatment with RNase R and actinomycin D, it was observed that circPCNXL2 exhibited greater stability compared to linear PCNXL2. (Fig. 1h-i). These results demonstrated the circular characteristics of circPCNXL2. The circPCNXL2 transcript was mainly in the cytoplasm of HuCCT1 and RBE by fluorescence in situ hybridization (FISH) and cytoplasmic and nuclear RNA purification assays (Fig. 1j-l). The above finding suggests that circPCNXL2 is overexpressed in ICC tissues and mainly located in the cytoplasm of ICC cells.

### CircPCNXL2 promotes the proliferation and migration of ICC in vitro

**Fig. 2:**
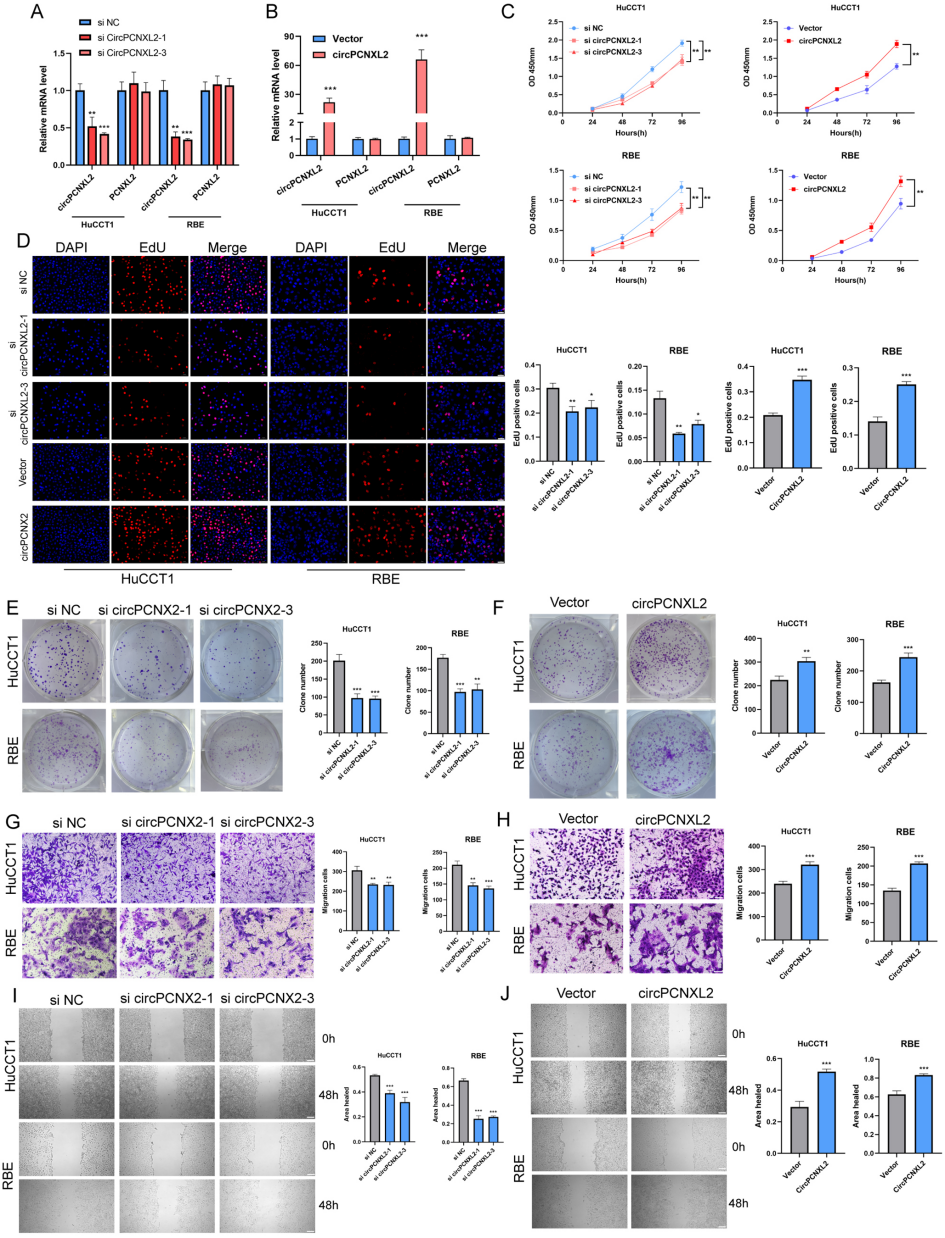
CircPCNXL2 promotes the proliferation and migration of ICC in vitro. **(a-b)** The efficiency of circPCNXL2 siRNAs or circPCNXL2 plasmid was certified by qRT-PCR. **(c)** CCK8 assay was performed in HuCCT1 and RBE cells transfected with circPCNXL2 siRNAs or circPCNXL2 plasmid. **(d)** EdU assay was conducted to evaluate the proliferation in HuCCT1 and RBE cells, scale bar = 50 μm. **(e-f)** Colony formation assay of HuCCT1 and RBE cells. **(g-h)** Transwall assay was performed in HuCCT1 and RBE cells transfected with circPCNXL2 siRNAs or circPCNXL2 plasmid, scale bar = 100 μm. **(i-j)** Wound healing assay was used to evaluate the migration in HuCCT1 and RBE cells, scale bar = 100 μm. *P < 0.05, **P < 0.01, ***P < 0.001

To elucidate the biological function of circPCNXL2 in ICC, siRNAs targeting the splice junction were used to knockdown the expression of circPCNXL2, and the circPCNXL2 plasmid was synthesized to overexpress circPCXNL2. The efficiency of siRNAs and plasmids was verified by qRT-PCR (Fig. 2a-b).

Firstly, the effect of circPCNXL2 on cell proliferation was measured by CCK-8, EdU assays, and clone formation assays. The results demonstrated that circPCNXL2 knockdown suppressed the proliferation of HuCCT1 and RBE cells. In contrast, the opposite effects were found in the circPCNXL2 overexpression group (Fig. 2c-f). Consistently, the ability of migration was impeded by circPCNXL2 knockdown and promoted by overexpressing circPCNXL2 by transwell assay and wound healing assay (Fig. 2g-j). These findings indicated the oncogenic role of circPCNXL2 in ICC cells.

### CircPCNXL2 facilitates the tumor growth and metastasis of ICC in vivo

**Fig. 3:**
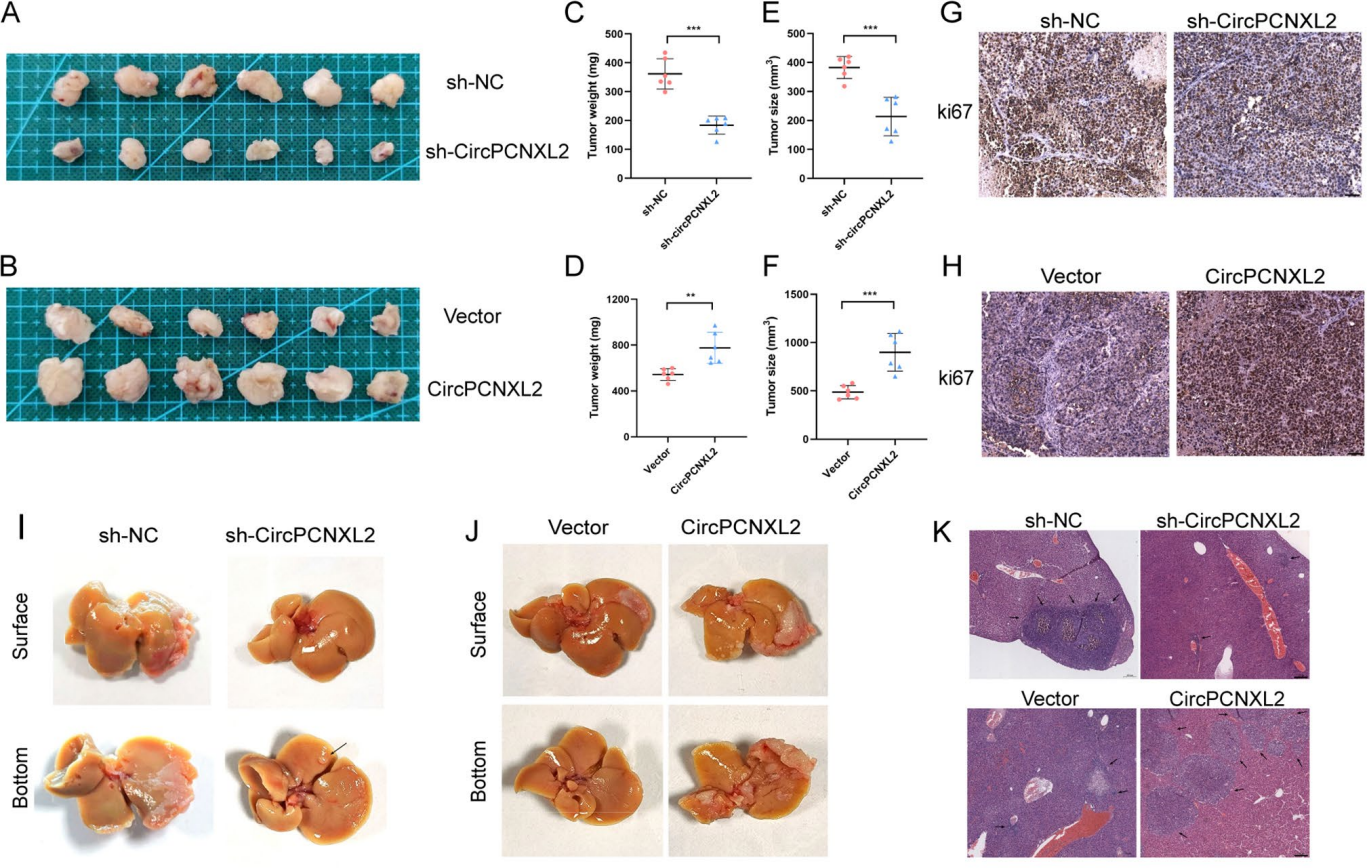
CircPCNXL2 facilitates the tumor growth and metastasis of ICC in vivo **(a-b)** Representative images of subcutaneous xenograft tumors (*n* = 6) **(c-f)** Weight and Volume of subcutaneous xenograft tumors show the effect of circPCNXL2 knockdown or overexpression on the formation of ICC. **(g-h)** Ki-67 staining of subcutaneous xenograft tumors, scale bar = 50 μm. **(i-j)** Representative images of liver metastasis model in mice inoculated with circPCNXL2 knockdown or overexpression ICC cells. **(k)** HE staining of liver metastasis model, scale bar = 200 μm. *P < 0.05, **P < 0.01, ***P < 0.001

To elucidate the effect of circPCNXL2 on ICC in vivo, stable HuCCT1 transfected with sh-circPCNXL2 and the corresponding control sh-NC, as well as RBE cells with stable circPCNXL2 overexpression and the corresponding control NC, were subcutaneously inoculated into nude mice (Fig. S2a-b). In the xenograft models, the circPCNXL2 knockdown tumors were smaller in volume than the control group, while the tumors generated from RBE cells with circPCNXL2 overexpression had a faster growth than those in the control group (Fig. 3a-f). A weaker Ki-67 level was observed by Immunohistochemistry (IHC) staining in the circPCNXL2 knockdown group. Conversely, the overexpression of circPCNXL2 led to a stronger Ki-67 staining, suggesting a correlation between Ki-67 and circPCNXL2 expression levels (Fig. 3g-h). Therefore, the results suggested that circPCNXL2 promoted ICC proliferation in vivo.

Then, we constructed the liver metastasis model to examine the metastatic characteristics of circPCNXL2 in ICC in vivo. The results demonstrated that the number of metastatic nodules in livers was significantly reduced in mice that were injected with HuCCT1 cells with circPCNXL2 knockdown. And, the ability of hepatic dissemination was enhanced by circPCNXL2 overexpression (Fig. 3i-k). These findings revealed that circPCNXL2 facilitated metastasis of ICC cells in vivo.

### CircPCNXL2 modulates the activation of the ERK/MAPK signaling pathway in ICC

**Fig. 4:**
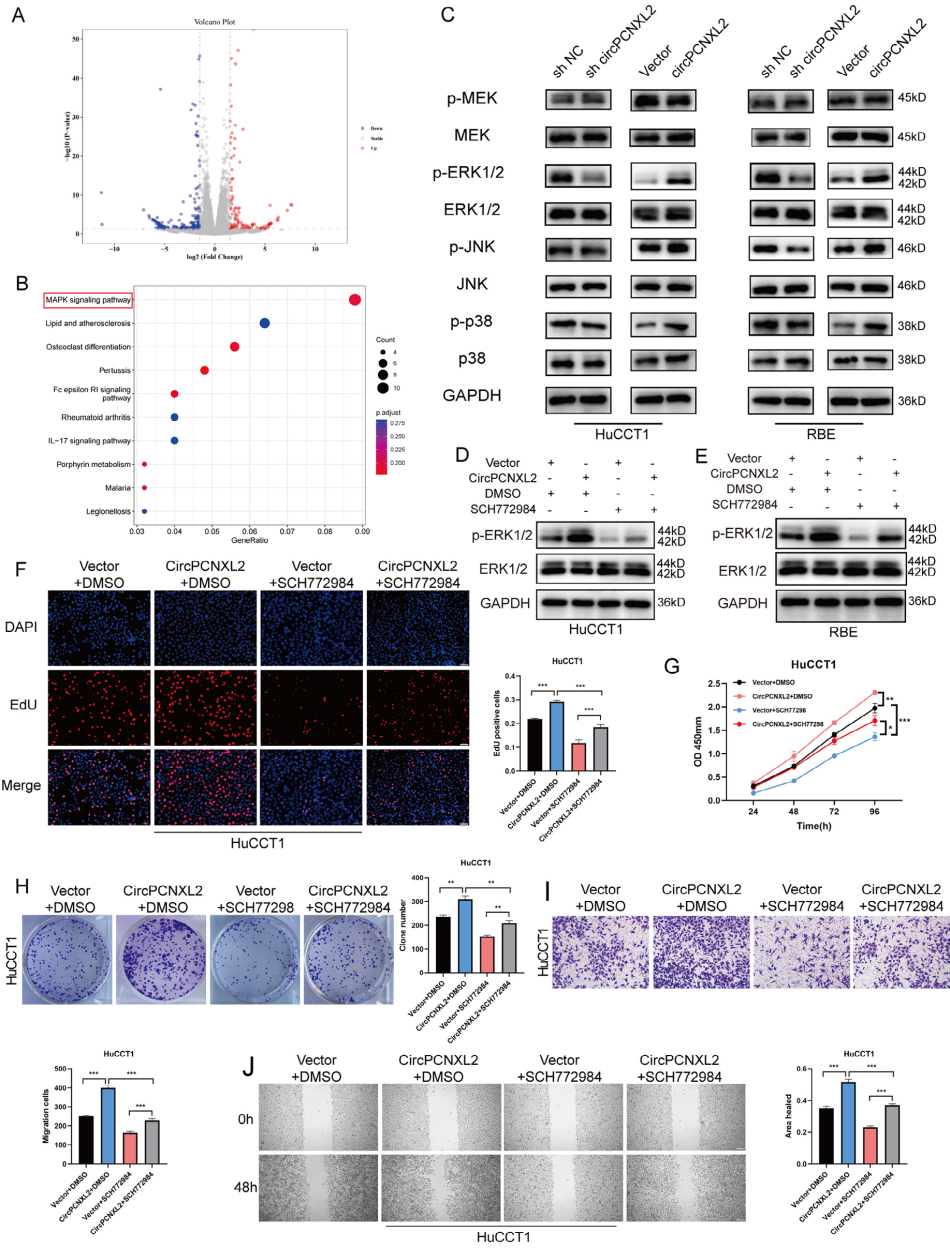
CircPCNXL2 modulates the activation of ERK/MAPK signaling pathway in ICC. **(a)** Volcano plot of the differentially expressed genes in circPCNXL2 overexpression group and control group. **(b)** KEGG pathway analysis of differentially expressed genes. **(c)** Western Bolt analysis showed the level of three subgroups of the MAPK signaling pathway in HuCCT1 and RBE cells. **(d-e)** Western blot analysis of the p-ERK and ERK in HuCCT1 and RBE cells. **(f)** EdU assay was conducted to evaluate the proliferation in HuCCT1 cells, scale bar = 50 μm. **(g)** CCK8 assay of HuCCT1 cells. **(h)** Colony formation assay of HuCCT1 cells. **(i)** Wound healing assay was used to evaluate the migration in HuCCT1 cells, scale bar = 100 μm. **(j)** Transwall assay was performed in HuCCT1 and RBE cells transfected with circPCNXL2 siRNAs or circPCNXL2 plasmid, Scale bar = 100 μm. *P < 0.05, **P < 0.01, ***P < 0.001

To dissect the potential mechanism of circPCNXL2 in promoting ICC cell proliferation and metastasis, we identified 278 differentially expressed genes (DEGs) in RBE cells overexpressing circPCNXL2 by RNA-seq (*p* < 0.05 and log |FC|>1.5, Fig. 4a). Kyoto Encyclopedia of Genes and Genomes (KEGG) pathway analysis revealed that the MAPK signaling pathway was the most enriched pathway (Fig. 4b). Gene Ontology (GO) enrichment analysis indicated that circPCNXL2 may regulate ERK1 and ERK2 cascade, a subgroup of MAPK pathway, in the progression of ICC (Fig. S3a). MAPK signaling pathway is reported to modulate several critical cellular functions such as cell proliferation and migration [21]. Based on this, we hypothesized that circPCNXL2 may promote the growth and metastasis of ICC through the MAPK pathway. As shown in immunoblotting results, the phosphorylation level of three subgroups in MAPK pathways were all enhanced in circPCNXL2 overexpression in HuCCT1 and RBE cells and all decreased in circPCNXL2 knockdown groups (Fig. 4c). The change of ERK phosphorylation was most remarkable in three subgroups, aligning with the result of GO enrichment. Consequently, we assumed that circPCNXL2 promoted the tumorigenesis of ICC mainly via the ERK/MAPK signaling pathway.

To comprehend the significance of ERK/MAPK pathway in the progression of ICC mediated by circPCNXL2, SCH772984, a highly selective ERK inhibitor, was added in vitro to block the phosphorylation of ERK. We verified that SCH772984 could effectively inhibit the activity of the ERK pathway by Western Blot (Fig. 4d-e). Functionally, EdU assays, CCK8 assays, Clone formation assay, transwell assay, and wound healing assay demonstrated that circPCNXL2 overexpression promoted proliferation and migration, which could be abrogated by SCH772984 (Fig. 4f-j and Fig. S3b-f). These results collectively suggest that circPCNXL2 facilitated the growth and migration of ICC via activation of the ERK/MAPK signaling pathway.

### CircPCNXL2 interacts with STRAP to mediate ERK/MAPK signaling pathway activation

**Fig. 5:**
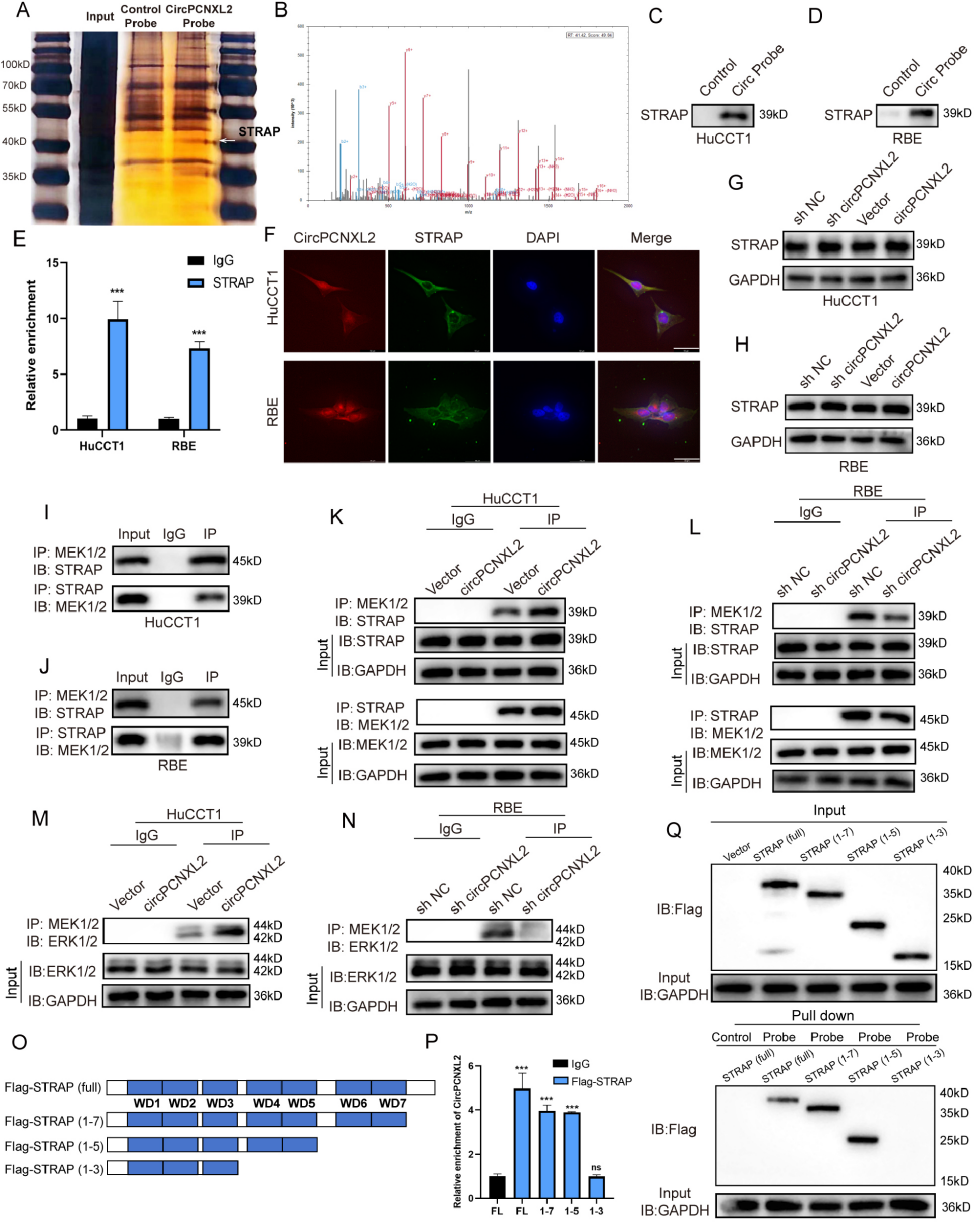
CircPCNXL2 interacts with STRAP to mediate ERK/MAPK signaling pathway activation. **(a)** CircPCNXL2 probe and control probe were applied for RNA pull-down assays in RBE cells, followed by silver staining. A specific band, indicated by the arrow, appeared at approximately 40 kD. **(b)** The typical STRAP peptide was identified in circPCNXL2-enriched proteins based on MS analysis. **(c-d)** Western blot showed the levels of STRAP were detected in proteins pulled down by circPCNXL2 probe or control probe in HuCCT1 and RBE cells. **(e)** RIP assays were performed using STRAP or IgG antibodies. **(f)** The localization of circPCNXL2 and STRAP were detected by FISH assay and immunofluorescence assays, scale bar = 50 μm. **(g-h)** The protein levels of STRAP were examined by Western blot in circPCNXL2 overexpression or knockdown HuCCT1 and RBE cells. **(i-j)** The binding of MEK1/2 to STRAP was confirmed by co-IP with STRAP or MEK1/2 antibody. **(k-l)** Co-IP assay was applied to investigate the interaction between MEK1/2 and STRAP in the HuCCT1 and RBE cells transfected with circPCNXL2 overexpression or circPCNXL2 knockdown. **(m-n)** Co-IP assay was used to detect the interaction between MEK1/2 and STRAP in the HuCCT1 and RBE cells. **(o)** The diagrams of Flag-tagged STRAP truncations mutants. **(p)** RIP assays were used to detect the enrichment of circPCNXL2 in 293T cells transfected with full-length and truncated Flag-tagged constructs mutants. **(q)** co-IP assay was performed in 293T cells transfected either with indicated vectors to identify the binding domain of STRAP responsible for its interaction with circPCNXL2. *P < 0.05, **P < 0.01, ***P < 0.001

To investigate how circPCNXL2 regulates the phosphorylation of ERK in ICC, biotinylated probe of circPCNXL2 was employed for RNA pull-down in RBE lysates, followed by silver staining assays and mass spectrometry (MS) analysis. Then, we observed a specific band at approximately 40 KD and the results of MS showed that STRAP may be able to bind to circPCNXL2 (Fig. 5a-b). We next confirmed the interaction between circPCNXL2 and STRAP in HuCCT1 and RBE cells by RNA pull-down and RIP assays (Fig. 5c-e). And we confirmed the co-localization of circPCNXL2 and STRAP by FISH assay and immunofluorescence (Fig. 5f). Interestingly, circPCNXL2 knockdown or overexpression did not exert the effect on the expression of STRAP (Fig. 5g-h). We supposed that circPCNXL2 may act as scaffolding in promoting ERK activation. STRAP can interact with MEK1/2 and is involved in the interaction between MEK1/2 and ERK1/2, which is required for the phosphorylation of the ERK pathway in colon cancer [15]. To test whether STRAP interacts with MEK1/2 in ICC, we performed immunoprecipitation and Western Blot assays in HuCCT1 and RBE. The results demonstrated that MEK1/2 was co-immunoprecipitated by anti-STRAP antibodies, and STRAP was also co-immunoprecipitated by anti-MEK1/2 antibodies. The finding indicated that STRAP could bind to MEK1/2 in ICC cells (Fig. 5i-j). Then, to investigate whether circPCNXL2 has any effect on the interaction between STRAP and MEK1/2 in ICC, we conducted immunoprecipitation assays with STRAP or MEK1/2 antibodies in circPCNXL2 overexpression or knockdown in both HuCCT1 and RBE cells. We observed that circPCNXL2 overexpression could promote the association between STRAP and MEK1/2 with no change in the level of their protein, on the contrary, circPCNXL2 knockdown attenuated the STRAP-MEK1/2 interaction in ICC cells (Fig. 5k-l). Moreover, the further result of immunoprecipitation assays with MEK1/2 antibodies confirmed that circPCNXL2 was also involved in the ERK1/2-MEK1/2 interaction and promoted the phosphorylation of ERK1/2 in both HuCCT1 and RBE cells (Fig. 5m-n). These data explained that, in ICC, circPCNXL2 could interact with STRAP and then promote the association between STRAP and MEK1/2, which is responsible for the phosphorylation of ERK1/2 by MEK1/2.

Furthermore, to dissect the precise binding domain of the interaction between circPCNXL2 and STRAP, a series of Flag-tagged STRAP deletion mutants were designed for RIP and RNA pull-down assays. The results revealed that the fourth and fifth WD-40 domain is responsible for the binding of STRAP with circPCNXL2 (Fig. 5o-q).

### CircPCNXL2 upregulates SRSF1 expression by sponging miR-766-3p

**Fig. 6:**
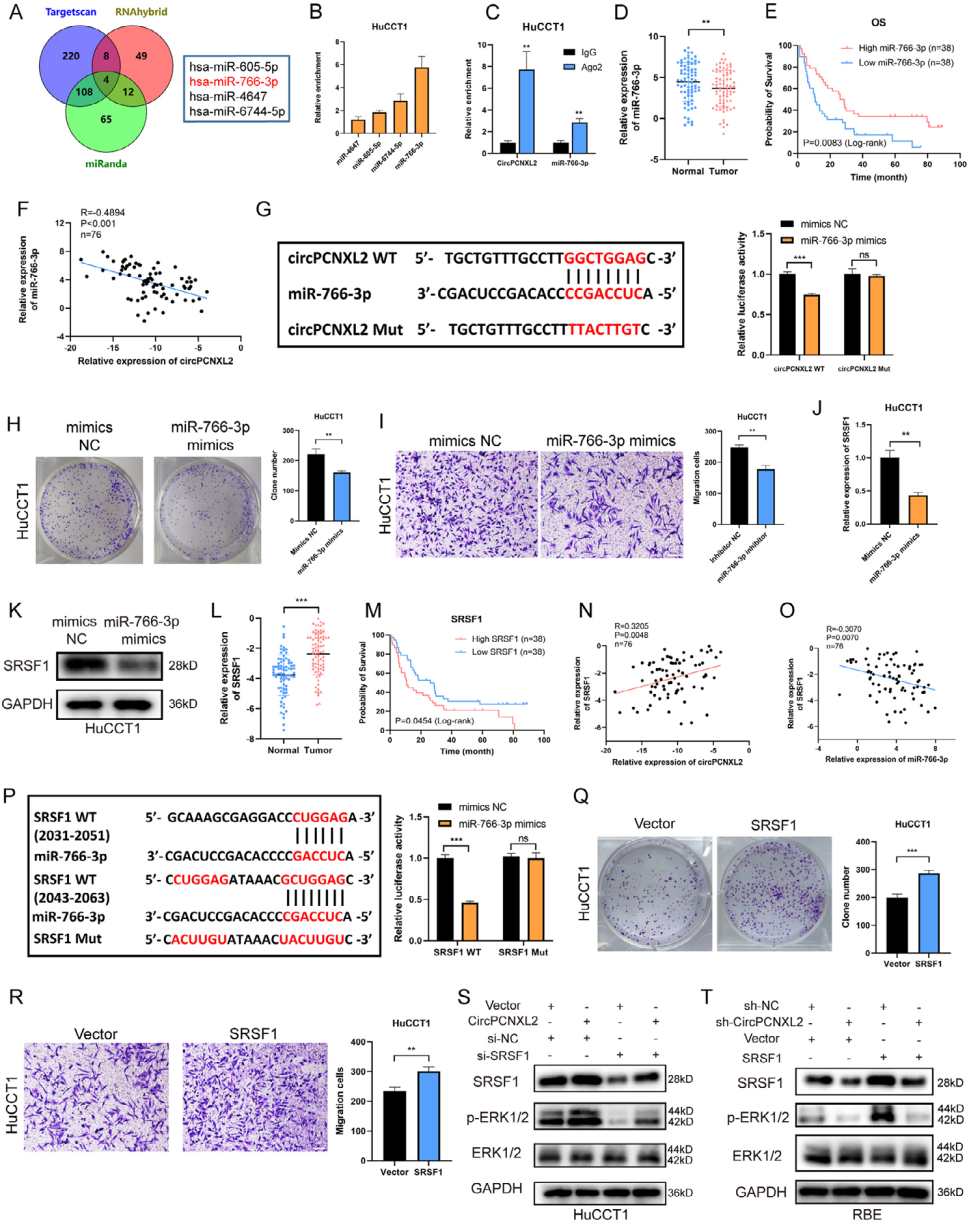
CircPCNXL2 upregulates SRSF1 expression by sponging miR-766-3p. **(a)** Venn diagram showed the overlap of the target miRNAs of circPCNXL2 predicted by Targetscan, miRanda and RNAhybrid. **(b)** The enrichment of 4 putative miRNAs was evaluated in the RNA pull down by circPCNXL2 probe in HuCCT1 cells. **(c)** The enrichment of circPCNXL2 and miR-766-3p was confirmed by RIP with AGO2 or IgG antibody in HuCCT1 cells. **(d)** The expression of miR-766-3p in 76 paired ICC tissues was measured by qRT-PCR. **(e)** Kaplan–Meier analysis of miR-766-3p expression and overall survival in 76 patients with ICC. The cutoff is the median expression value of miR-766-3p. **(f)** Correlation analysis showed a negative relationship between the levels of circPCNXL2 and miR-766-3p. **(g)** The wild-type (WT) and mutant (Mut) circPCNXL2 plasmid for dual-luciferase report were applied to confirm the interaction between circPCNXL2 and miR-766-3p. **(h)** The functions of miR-766-3p on the proliferation of HuCCT1 cells were detected by colony formation assay. **(i)** The functions of miR-766-3p on the migration of HuCCT1 cells was detected by transwell assay, scale bar = 100 μm. **(j-k)** The mRNA and protein levels of SRSF1 were evaluated in HuCCT1 transfected with miR-766-3p mimics. **(l)** The expression of SRSF1 in 76 paired ICC tissues was measured by qRT-PCR. **(m)** Kaplan–Meier analysis of SRSF1 expression and overall survival in 76 patients with ICC. The cutoff is the median expression value of SRSF1. **(n-o)** Correlation analysis showed a negative relationship between the levels of SRSF1 and miR-766-3p and a positive relationship between the levels of SRSF1 and circPCNXL2. **(p)** The wild-type (WT) and mutant (Mut) SRSF1 plasmid for dual-luciferase report were applied to confirm the interaction between miR-766-3p and SRSF1. **(q)** The functions of SRSF1 on the proliferation of HuCCT1 cells was detected by colony formation assay. **(r)** The functions of SRSF1 on the migration of HuCCT1 cells was detected by transwell assay, scale bar = 100 μm. **(s-t)** The levels of SRSF1, p-ERK and ERK were measured in HuCCT1 or RBE cells co-transfected with circPCNXL2 overexpression and/or SRSF1 knockdown. *P < 0.05, **P < 0.01, ***P < 0.001

CircRNAs are reported that they may exert functions in many other manners, such as encoding functional peptides and acting as miRNA sponges [22, 23]. However, none of the putative open reading frames (OFR) was annotated in circRNADb [24], which indicated that circPCNXL2 may not be able to encode protein. Additionally, RNA immunoprecipitation (RIP) showed that circPCNXL2 was remarkably enriched by Argonaute 2 (AGO2) antibody in HuCCT1 and RBE cells, suggesting that circPCNXL2 may function as a miRNA inhibitor in ICC. Then, we identified 4 candidate miRNAs (miR-605-5p, miR-766-3p, miR-4647, and miR-6744-5p) by overlapping the miRNA target prediction by Targetscan, miRanda, and RNAhybrid [25–27] (Fig. 6a). Next, RNA pull-down assays with biotin-labeled probe of circPCNXL2 was used to bind to potential miRNAs and miR-766-3p was the most highly enriched miRNA in both HuCCT1 and RBE cells (Fig. 6b and Fig. S4a). The results of RIP assays indicated that the miR-766-3p was enriched by AGO2 antibody rather than IgG antibody (Fig. 6c and Fig. S4b). Then we confirmed miR-766-3p is downregulated in ICC tissues compared to normal tissues and a low level of miR-766-3p in ICC patients was associated with poor survival (Fig. 6d-e). Furthermore, we observed that the expression of miR-766-3p in ICC tissues was negatively correlated with circPCNXL2 levels (Fig. 6f).

In Addition, we designed the mutant plasmid of circPCNXL2 according to the binding regions between circPCNXL2 and miR-766-3p, and the result of a dual-luciferase report assay demonstrated that luciferase activity was remarkably decreased in co-transfected wild type circPNCXL2 and miR-766-3p mimics compared with that in control group, while no difference was found in mutant circPCNXL2 group (Fig. 6g). These data revealed that circPCNXL2 acts as a miR-766-3p sponge in ICC.

To investigate the functions of miR-766-3p in ICC growth, miR-766-3p mimics or inhibitor was transfected in HuCCT1 or RBE cells (Fig. S4c-d). The results indicated that miR-766-3p mimics significantly suppressed the proliferation and migration in HuCCT1 cells and miR-766-3p inhibitor promoted ICC progression in RBE cells. (Fig. 6h-i and Fig.S4e-f). Collectively, these findings showed that miR-766-3p could inhibit the oncogenesis of ICC cells.

In the previous study, miR-766-3p could suppress the progression of renal cell cancer by targeting SRSF1 directly [28]. Therefore, we assumed that SRSF1 also could be the downstream gene of miR-766-3p in ICC. The mRNA and protein level of SRSF1 was decreased in HuCCT1 cells transfected with miR-766-3p mimics, which was increased in RBE cells transfected with miR-766-3p inhibitor (Fig. 6j-k and Fig.S4g-h). However, the functions of SRSF1 in the progression of ICC are still unclear. Then, we examined the SRSF1 expression in tissues using qRT-PCR and we found SRSF1 is upregulated in ICC tissues compared to normal tissues, while similar results were observed in the TCGA database (Fig. 6l and Fig. S4i) [29]. The high expression of SRSF1 was an oncogenic factor for ICC patients (Fig. 6m). Additionally, the result of qRT-PCR indicated a positive correlation between SRSF1 expression and circPCNXL2 expression, while a negative association was observed with miR-766-3p expression (Fig. 6n-o). Then, we designed the mutant plasmid of SRSF1 according to the binding regions between SRSF1 and miR-766-3p, and the result of a dual-luciferase report assay confirmed the interaction between SRSF1 and miR-766-3p (Fig. 6p). To explore the functions of SRSF1 in ICC progression, SRSF1 overexpression and knockdown was constructed in HuCCT1 and RBE cells (Fig. S4j-k). The results of clone formation and transwell assays demonstrated that SRSF1 overexpression significantly facilitated the proliferation and migration in HuCCT1 cells. Opposite results were found in RBE cells which were transfected with SRSF1 knockdown (Fig. 6q-r and Fig. S4l-m). Moreover, SRSF1 was reported that it was required for the activation of ERK [30, 31], and we confirmed SRSF1 could promote the phosphorylation of ERK in HuCCT1 and RBE cells, which could be rescued by circPCNXL2 overexpression or knockdown (Fig. 6s-t). In vivo, we assessed the expression of miR-766-3p using qRT-PCR and measured the level of SRSF1 through IHC staining (Fig. S4n-p). The results were consistent with the findings observed in vitro. All these findings showed that circPCNXL2 could modulate the proliferation and migration of ICC via miR-766-3p/SRSF1/ERK pathway.

### CircPCNXL2 acts as a potential therapeutic target and alleviates the anti-tumor effects of trametinib in vivo

**Fig. 7:**
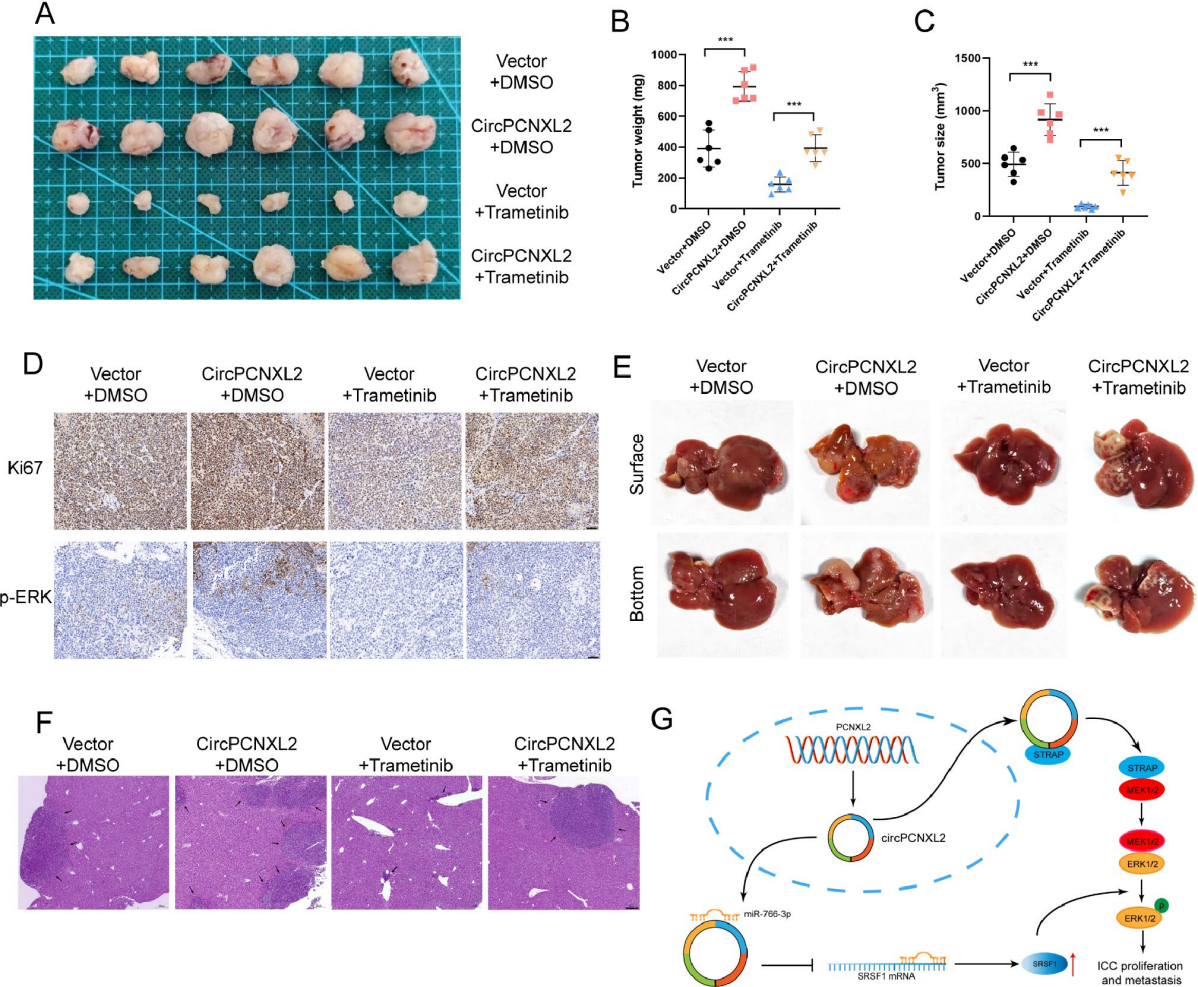
CircPCNXL2 acts as a potential therapeutic target and alleviates anti-tumor effects of trametinib in vivo. **(a)** Representative images of subcutaneous xenografts. HuCCT1 cells transfected with vector or circPCNXL2 plasmid were subcutaneously injected into the node mice. Mice were treated with or without 1 mg/kg trametinib daily gavage for 15 days. **(b-c)** Tumor weight and volume of subcutaneous xenografts. **(d)** IHC staining of Ki-67 and p-ERK in xenografts, scale bar = 50 μm. **(e)** Liver metastasis models were established by injecting with indicated cells. HuCCT1 cells transfected with vector or circPCNXL2 plasmid were subcutaneously injected into the node mice. Mice were treated with or without 1 mg/kg trametinib daily gavage for 15 days. **(f)** HE staining of liver metastasis model, scale bar = 200 μm. **(g)** Schematic diagram illustrating the mechanism by which circPCNXL2 promotes ICC proliferation and metastasis. CircPCNXL2 activates ERK phosphorylation by promoting interaction between MEK1/2 and ERK1/2 through direct binding to STRAP and the miR-766-3p/SRSF1 axis. *P < 0.05; **P < 0.01; ***P < 0.001

Trametinib, an orally MEK inhibitor, is approved to be used alone or in combination to treat melanoma, non-small-cell lung cancer and other cancers [32, 33]. Notably, a series of research on trametinib was conducted in ICC [34–36]. Therefore, to investigate the effects of circPCNXL2 on trametinib, trametinib was applied to inhibit the MEK/ERK pathway in vivo and HuCCT1 cells with circPCNXL2 overexpression were utilized to establish subcutaneous xenograft models and liver metastasis models. The experiment in vivo containing four groups (Vector + DMSO, circPCNXL2 OE + DMSO, Vector + trametinib, circPCNXL2 OE + trametinib) was conducted. In the xenograft models, the tumor growth was suppressed by trametinib, and this inhibitory effect was rescued by circPCNXL2 overexpression (Fig. 7a-c). IHC staining of xenografts revealed the staining of Ki-67 and p-ERK was weakest in the trametinib group (Fig. 7d). Upregulation of circPCNXL2 remarkably attenuated the anti-tumor effect of trametinib in ICC. Similar results were observed in liver metastasis models. The treatment with trametinib reduced the number of liver metastasis foci, which could be attenuated by overexpressing circPCNXL2 (Fig. 7e-f). Collectively, these data proved that circPCNXL2 inhibited the antineoplastic efficacy of trametinib in ICC by activating the phosphorylation of ERK in vivo, which could be a promising strategy in ICC therapy.

## Discussion

Non-coding RNAs (ncRNAs) represent a diverse group of heterogeneous transcripts and dysregulated expression of ncRNAs is recognized to play a pivotal role in the tumorigenesis of various cancers, including ICC [37, 38]. CircRNAs are a type of ncRNAs characterized by their closed circular structure, contributing to enhanced stability compared to linear RNA [22]. This unique feature has prompted the research into understanding the functions of circRNAs in ICC development and their potential as diagnostic and prognostic biomarkers. For instance, circHMGCS1–016 could promote ICC development by reshaping the immune environment via miR-1236-3p/CD73 and GAL-8 signaling [39]. CircCCAC1 promotes the progression of ICC by inducing angiogenesis, and disrupting vascular endothelial barriers via the miR-514a-5p/YY1/CAMLG axis [40]. In our present study, we first identified differentially expressed circRNAs in ICC by analyzing the data from the GSE181523 dataset and we found circPCNXL2 is upregulated in ICC tissues. Correlation analysis between the clinical pathological and circPCNXL2 expression indicated that a significant association between high circPCNXL2 expression and advanced TNM stage. Meanwhile, patients in the high circPCNXL2 group exhibited an unfavorable prognosis. In univariate and multivariate analyses, circPCNXL2 was identified as a risk factor for ICC patients. Functional experiments revealed the oncogenic role of circPCNXL2, as its overexpression promoted ICC cell growth and migration and its knockdown inhibited the progression of ICC in vitro. These results were consistently confirmed in vivo.

In our RNA-seq analysis comparing the overexpression of circPCNXL2 to the control group in RBE cells, we observed significant alterations in the MAPK signaling pathway Furthermore, GO enrichment analysis highlighted the involvement of ‘ERK1 and ERK2 cascade’ and ‘regulation of ERK1 and ERK2 cascade’. MAPK pathways are reported involved in all aspects of human life and altered in many diseases. MEK/ERK pathway, one of three subgroups within MAPK pathways, is reported that involved in the tumorigenesis of numerous cancers by facilitating tumor growth, metastasis, angiogenesis, and extracellular matrix degradation [41]. The dysregulation of the MEK/ERK pathway is found in 35% of ICC patients, and the activation of ERK is related to pro-malignant functions in ICC [42]. In our study, circPCNXL2 could play an essential role in the oncogenesis of ICC by facilitating the phosphorylation of ERK, instead of modulating MEK. Inhibiting the activation of ERK with SCH772984 (an ERK phosphorylation inhibitor) could partially abolish the oncogenesis of circPCNXL2 in ICC cells. In brief, we found that circPCNXL2 could promote ICC progression by modulating the MEK/ERK pathway. However, RNA-seq on ICC tissues and patient-derived tumor xenograft model was not conducted to explore the functions and mechanisms of circPCNXL2 in ICC. Similar investigations are warranted to elucidate the clinical value of circPCNXL2 in ICC in future studies.

CircRNAs are reported that they can exert functions in tumor growth by interacting with proteins in ICC. For example, circNFIB inhibits ICC growth and metastasis by interacting with MEK1 [43]. And a study showed that circMBOAT2 promoted ICC and metabolism reprogramming by interacting with PTBP1 [44]. Our study demonstrated that circPCNXL2 could directly bind to the fourth and fifth WD-40 domain of STRAP, interestingly, the expression of STRAP remained unchanged by circPCNXL2 overexpression or knockdown. In colon cancer, STRAP recruits MEK1/2 and enhances the interaction between MEK1/2 and ERK1/2 to activate the phosphorylation of ERK1/2 [15]. Our findings confirmed that circPCNXL2 enhanced the binding of STRAP to MEK1/2, resulting in the increased interaction between MEK1/2 and ERK1/2, ultimately promoting the activation of the MEK/ERK pathway.

The ceRNA network is the most popular mechanism for circRNAs to regulate tumor progression, and circRNAs can competitively combine with miRNA to upregulate its downstream mRNA [45]. For instance, circLMO7 acts as a miR-30a-3p sponge to promote the expression of WNT2 [46]. In our present study, we also found circPCNXL2 could act as a miR-766-3p sponge to upregulate SRSF1, facilitating ICC proliferation and migration via the MEK/ERK pathway. We performed a correlation analysis between the expression of miR-766-3p, SRSF1 and clinicopathological characteristics (Table S2). Unfortunately, due to the limited sample size, meaningful results were not observed. Thus, to further validate the association between circACTN4 expression (or the target genes of circPCNXL2) and disease prognosis, a larger sample size will be required in the future studies.

ICC is an aggressive cancer and diagnosing at an early stage remains a challenge because most ICC patients are asymptomatic [3]. Radical operation is the preferred curative treatment option for ICC patients, but only almost 35% of patients at an early stage have a chance to receive surgical resection [2]. For the unresectable patients, the median overall survival with the standard chemotherapy treatment is almost 1 year in a few years ago [47]. However, with the deep studies of ICC in recent years, many clinical trials of targeted therapy and immunotherapy have been undergone. In a phase II study, the progression-free survival in patients treated with ivosidenib (an inhibitor of mutant IDH1) is significantly improved compared to those with placebo (2.7 months versus 1.4 months) [48]. And another phase III study showed durvalumab plus chemotherapy (TOPAZ-1) significantly improved overall survival and median progression-free survival versus placebo plus chemotherapy [49]. In BRAF^V600F^-mutated biliary tract cancer, dabrafenib (a BRAF inhibitor) plus trametinib combination treatment may be an effective treatment option [34]. In mice ICC models, trametinib combined with anti-PD-1 can inhibit tumor growth and improve survival [36].

Our data showed that circPCNXL2 overexpression suppresses the anti-tumor efficiency of trametinib in vivo, indicating that circPCNXL2 may be a potential target for enhancing the activity of trametinib in ICC. However, further studies are required to determine the therapeutic value of trametinib and circPCNXL2 in ICC treatment and the systemic treatment for ICC remains several important questions that need to be addressed.

## Conclusion

In summary, our study reveals that circPCNXL2 is upregulated in ICC and plays a tumor promoter in ICC development by interacting with STRAP to promote the phosphorylation of ERK. Besides, circPCNXL2 acts as a sponge of miR-766-3p, leading to the upregulation of SRSF1, which further activates the MEK/ERK pathway in ICC (Fig. 7g). Collectively, our findings demonstrate that circPCNXL2 may serve as a diagnostic and prognostic biomarker for ICC patients. Moreover, the circPCNXL2-STRAP-MEK-ERK axis is a promising translational treatment target for further investigation in ICC.

### Electronic supplementary material

Below is the link to the electronic supplementary material.

Supplementary Material 1

Supplementary Material 2

Supplementary Material 3

Supplementary Material 4

Supplementary Material 5

Supplementary Material 6

Supplementary Material 7

Supplementary Material 8

Supplementary Material 9

Supplementary Material 10

Supplementary Material 11

Supplementary Material 12

## Data Availability

The datasets analyzed during the current study are available in the GEO repository (https://www.ncbi.nlm.nih.gov/geo, GEO accession number: GSE181523), TCGA repository (https://www.cancer.gov/tcga).

## Abbreviations

AGO2: Argonaute 2
CircRNAs: Circular RNAs
DAPI: 4’,6-diamidino-2-phenylindole
DEG: Differentially Expressed Genes
ERK: Extracellular Signaling-regulated Kinase
FISH: Fluorescence in Situ Hybridization
GEO: Gene Expression Omnibus
HCC: Hepatocellular Carcinoma
ICC: Intrahepatic Cholangiocarcinoma
IHC: Immunohistochemistry
JNK: Jun N-terminal kinases
KEGG: Kyoto Encyclopedia of Genes Genomes
LncRNAs: Long non-coding RNAs
miRNAs: MicroRNAs
MS: Mass Spectrometry
MAPK: Mitogen-Activated Protein Kinase
MVI: Microvascular invasion
ncRNAs: Non-coding RNAs
OFR: Open Reading Frame
STRAP: Serine/Threonine Kinase Receptor Associated Protein
SRSF1: Serine and Arginine Rich Splicing Factor 1
TCGA: The Cancer Genome Atlas
RIP: RNA immunoprecipitation
