# Supplementary figures and images for: CircPCNXL2 promotes tumor growth and metastasis by interacting with STRAP to regulate ERK signaling in intrahepatic cholangiocarcinoma
Source: Mol Cancer. 2024 Feb 17;23:35. doi: 10.1186/s12943-024-01950-y (PMC10873941; doi:10.1186/s12943-024-01950-y)

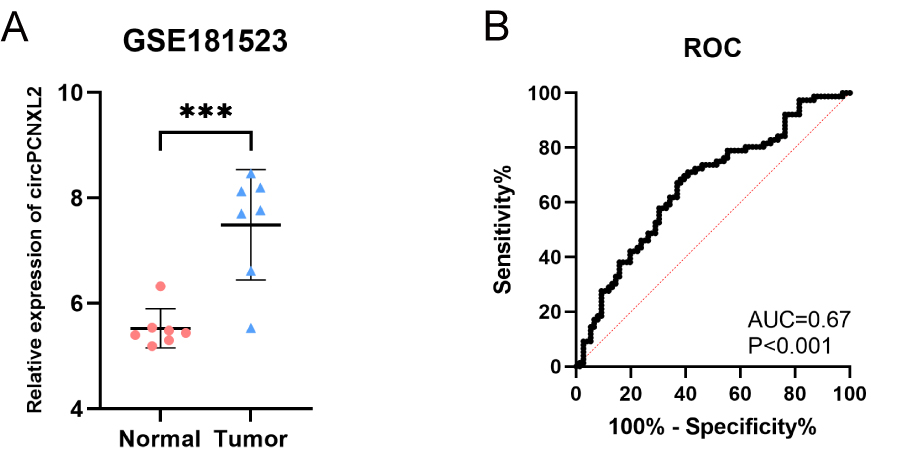

Supplement: Supplementary file 3 — Supplementary Material 3 [file 12943_2024_1950_MOESM3_ESM.jpg]

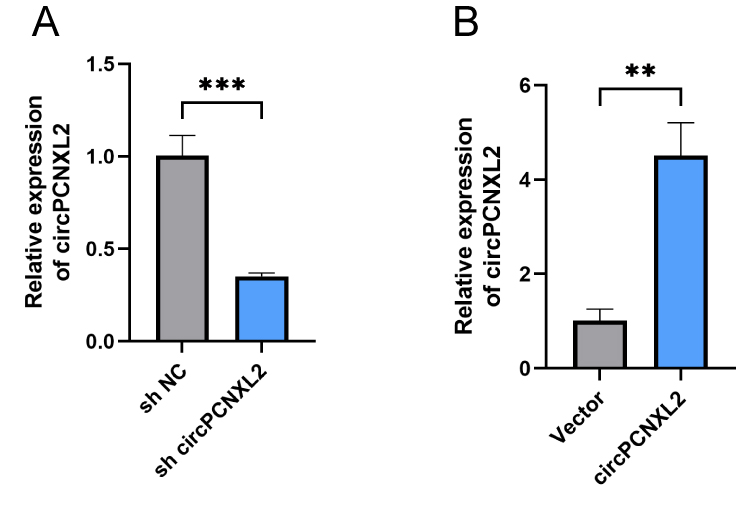

Supplement: Supplementary file 5 — Supplementary Material 5 [file 12943_2024_1950_MOESM5_ESM.jpg]

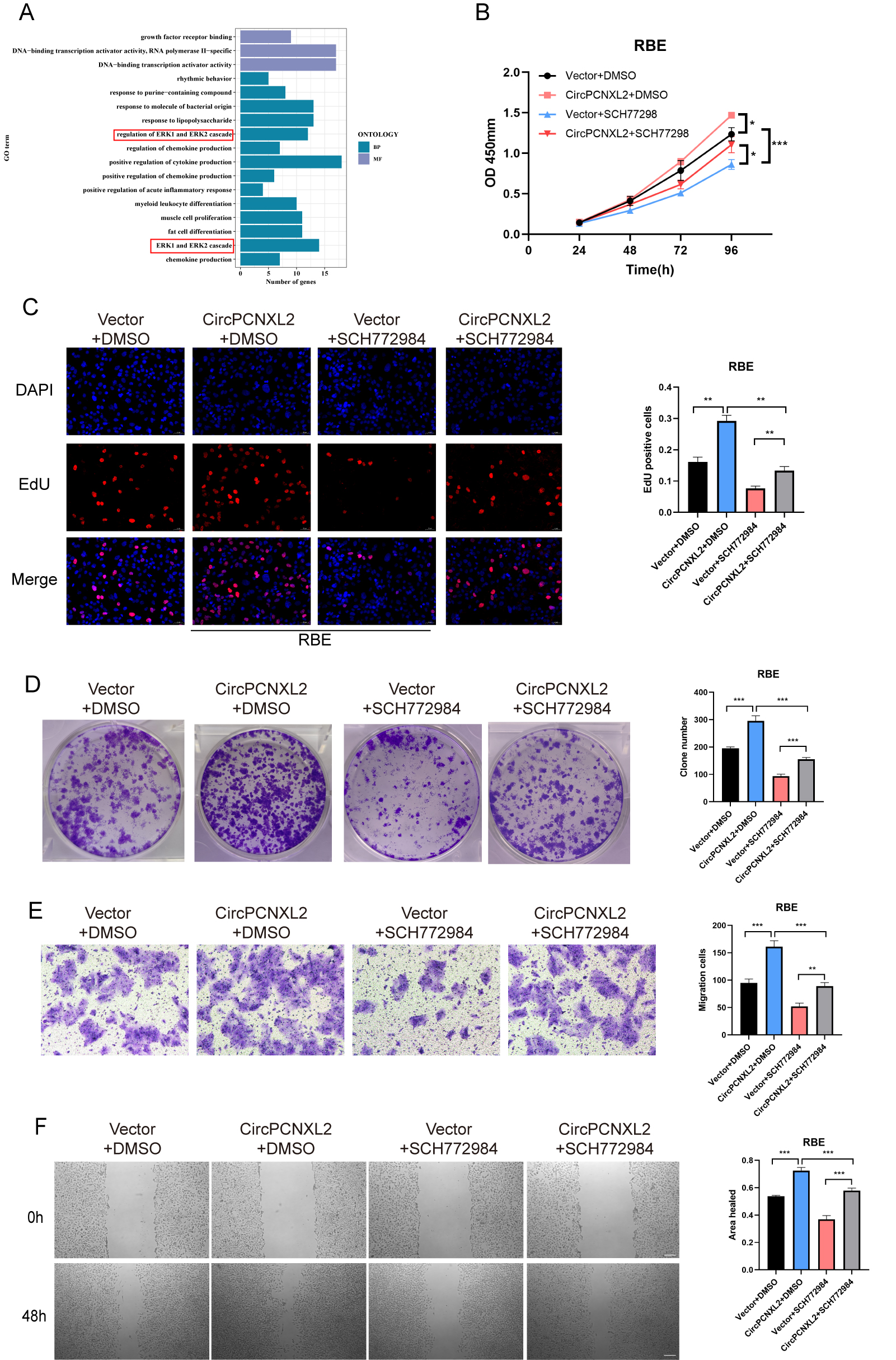

Supplement: Supplementary file 10 — Supplementary Material 10 [file 12943_2024_1950_MOESM10_ESM.jpg]

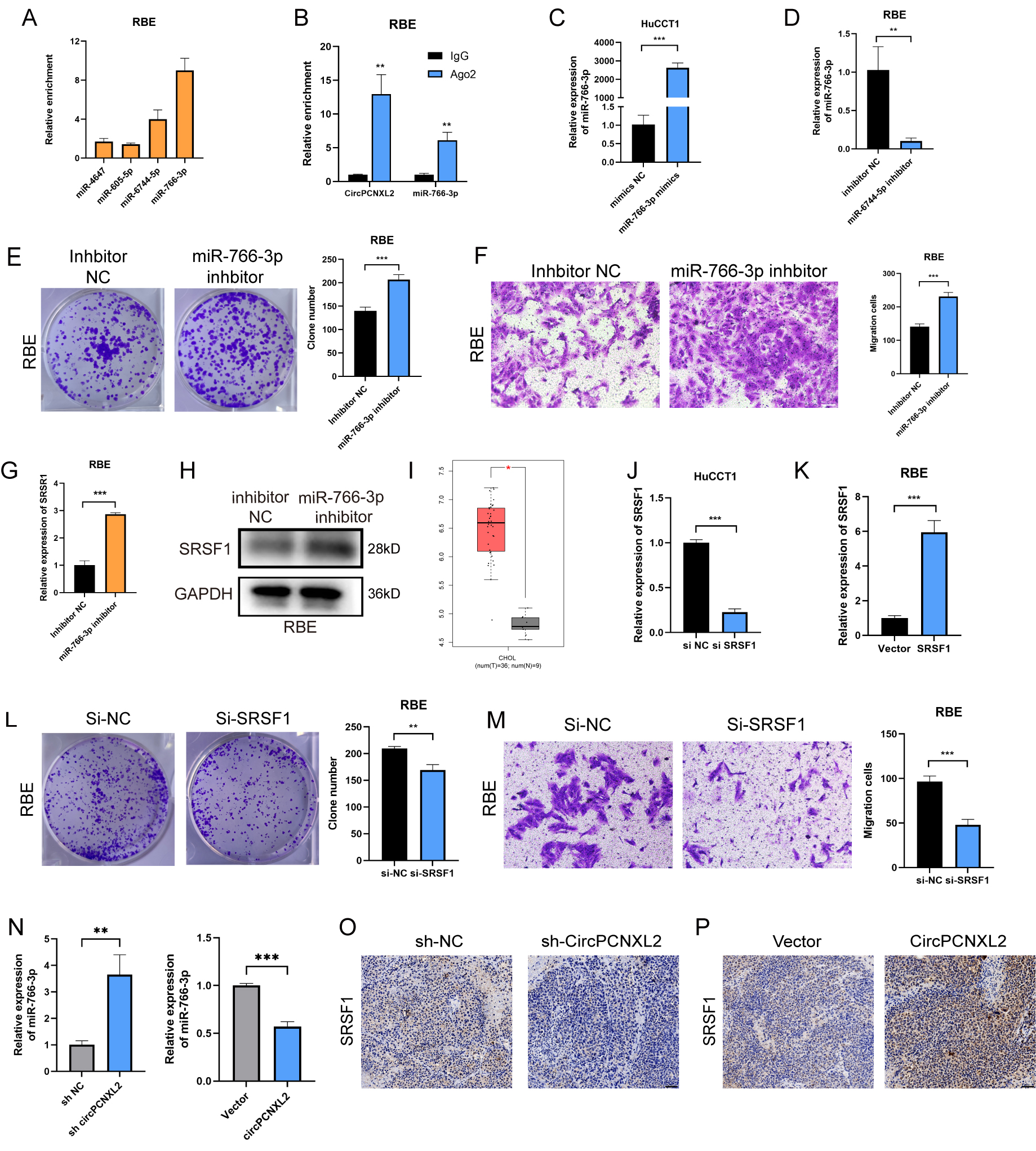

Supplement: Supplementary file 11 — Supplementary Material 11 [file 12943_2024_1950_MOESM11_ESM.jpg]
