# Supplementary material for: CircPCNXL2 promotes tumor growth and metastasis by interacting with STRAP to regulate ERK signaling in intrahepatic cholangiocarcinoma
Source: Mol Cancer. 2024 Feb 17;23:35. doi: 10.1186/s12943-024-01950-y (PMC10873941; doi:10.1186/s12943-024-01950-y)
Supplement: Supplementary file 6 — Supplementary Material 6 [file 12943_2024_1950_MOESM6_ESM.docx]

|  | **miR-766-3p expression** | |  | **SRSF1 expression** | |  |
| --- | --- | --- | --- | --- | --- | --- |
| **Characteristics** | **High**  **(N=38)** | **Low**  **(N=38)** | **P value** | **High**  **(N=38)** | **Low**  **(N=38)** | **p value** |
| Gender |  |  | 0.159 |  |  | 0.348 |
| Male | 26 | 20 |  | 21 | 25 |  |
| Female | 12 | 18 |  | 17 | 13 |  |
| Age |  |  | 0.247 |  |  | 0.488 |
| <60 | 24 | 19 |  | 23 | 20 |  |
| ≥60 | 14 | 19 |  | 15 | 18 |  |
| T stage |  |  | 0.791 |  |  | 0.427 |
| T1 | 28 | 29 |  | 30 | 27 |  |
| T2 - T4 | 10 | 9 |  | 8 | 11 |  |
| N stage |  |  | 0.128 |  |  | 0.361 |
| N0 | 34 | 29 |  | 30 | 33 |  |
| N1 | 4 | 9 |  | 8 | 5 |  |
| TNM stage |  |  | 0.150 |  |  | 0.150 |
| Ⅰ-Ⅱ | 33 | 28 |  | 28 | 33 |  |
| Ⅲ | 5 | 10 |  | 10 | 5 |  |
| HBsAg |  |  | 0.479 |  |  | 0.479 |
| Positive | 13 | 16 |  | 13 | 16 |  |
| Negative | 25 | 22 |  | 25 | 22 |  |
| Microvascular invasion |  |  | 0.773 |  |  | 0.150 |
| Yes | 7 | 8 |  | 5 | 10 |  |
| No | 31 | 30 |  | 33 | 28 |  |

**Table S2 Relationship between miR-766-3p, SRSF1 and clinicopathological characteristics in ICC patients.**
