# Supplementary material for: CircPCNXL2 promotes tumor growth and metastasis by interacting with STRAP to regulate ERK signaling in intrahepatic cholangiocarcinoma
Source: Mol Cancer. 2024 Feb 17;23:35. doi: 10.1186/s12943-024-01950-y (PMC10873941; doi:10.1186/s12943-024-01950-y)
Supplement: Supplementary file 7 — Supplementary Material 7 [file 12943_2024_1950_MOESM7_ESM.docx]

**Table S4 Antibodies and reagents used in this study.**

| Name | Company | Cat no. |
| --- | --- | --- |
| Anti-JNK | Abcam | ab199380 |
| Anti-p-JUK | Abcam | ab215208 |
| Anti-p38 | Abcam | ab170099 |
| Anti-p-p38 | Abcam | ab195049 |
| Anti-ERK | Abcam | ab184699 |
| Anti-Phospho-p44/42 MAPK (Erk1/2) | Cell Signaling Technology | 4370 |
| Anti-STRAP | Proteintech | 18277-1-AP |
| Anti-MEK1/2 | Proteintech | 11049-1-AP |
| Anti-Phospho-MAP2K1-S217/MAP2K2-S221 | Abclonal | AP0209 |
| Anti-SRSF1 | Abclonal | A4091 |
| Anti-GAPDH | Servicebio | GB15004-100 |
| Anti-Flag | Cell Signaling Technology | 14793 |
| Anti-IgG Control | Proteintech | 30000-0-AP |
| Anti-IgG, HRP-linked Antibody | Cell Signaling Technology | 7074 |
| SCH772984 | MCE | [HY-50846](https://www.medchemexpress.cn/SCH772984.html) |
| Trametinib | MCE | [HY-10999](https://www.medchemexpress.cn/Trametinib.html) |
