# Supplementary material for: CircPCNXL2 promotes tumor growth and metastasis by interacting with STRAP to regulate ERK signaling in intrahepatic cholangiocarcinoma
Source: Mol Cancer. 2024 Feb 17;23:35. doi: 10.1186/s12943-024-01950-y (PMC10873941; doi:10.1186/s12943-024-01950-y)
Supplement: Supplementary file 8 — Supplementary Material 8 [file 12943_2024_1950_MOESM8_ESM.docx]

| **Characteristics** | **Univariable analysis** | | |  | **Multivariable analysis** | | |  |
| --- | --- | --- | --- | --- | --- | --- | --- | --- |
|  | **HR** | **95%CI** | **P value** | | **HR** | **95%CI** | **P value** | |
| Gender  (Female vs male) | 0.669 | 0.395-1.136 | 0.137 | |  |  |  | |
| Age  (≥60 vs <60) | 1.028 | 0.612-1.726 | 0.918 | |  |  |  | |
| T stage  (T2 - T4 vs T1) | 2.226 | 1.267-3.912 | **0.005**** | | 2.894 | 1.284-6.522 | **0.010*** | |
| N stage  (N1 vs N0) | 3.227 | 1.673-6.222 | **< 0.001***** | | 7.147 | 1.313-38.886 | **0.023*** | |
| TNM stage  (Ⅲ vs Ⅰ-Ⅱ) | 2.683 | 1.471-4.894 | **0.001**** | | 0.281 | 0.052-1.523 | 0.141 | |
| HbsAg  (positive vs negative) | 0.803 | 0.467-1.380 | 0.427 | |  |  |  | |
| MVI  (Yes vs No) | 1.980 | 1.070-3.663 | **0.030*** | | 1.050 | 0.502-2.200 | 0.896 | |
| CircPCNXL2 expression  (high vs low) | 2.395 | 1.426-4.021 | **< 0.001***** | | 3.031 | 1.704-5.393 | **< 0.001***** | |

**Table S1 Univariate and multivariate analyses of overall survival in 76 ICC patients.**

**HR: hazard ratio, CI: confidence interval, TNM: tumor node metastasis. Cox regression analysis, *p<0.05, **p<0.01, ***p<0.001**
