# Supplementary material for: CircPCNXL2 promotes tumor growth and metastasis by interacting with STRAP to regulate ERK signaling in intrahepatic cholangiocarcinoma
Source: Mol Cancer. 2024 Feb 17;23:35. doi: 10.1186/s12943-024-01950-y (PMC10873941; doi:10.1186/s12943-024-01950-y)
Supplement: Supplementary file 9 — Supplementary Material 9 [file 12943_2024_1950_MOESM9_ESM.docx]

| Name | Sense (5’-3’) |
| --- | --- |
| miR-766-3p-RT | GTCGTATCCAGTGCAGGGTCCGAGGTATTCGCACTGGATACGACGCTGAG |
| miR-766-3p-Forward | CGACTCCAGCCCCACAGC |
| miRNA-Reverse | AGTGCAGGGTCCGAGGTATT |
| miR-605-5p-RT | GTCGTATCCAGTGCAGGGTCCGAGGTATTCGCACTGGATACGACAGGAGA |
| miR-605-5p-Forward | CGTAAATCCCATGGTGCCT |
| miR-4647-RT | GTCGTATCCAGTGCAGGGTCCGAGGTATTCGCACTGGATACGACTTCCTC |
| miR-4647-Forward | CGGAAGATGGTGCTGTGCT |
| miR-605-5p-RT | GTCGTATCCAGTGCAGGGTCCGAGGTATTCGCACTGGATACGACAGGCCT |
| miR-605-5p-Forward | GCGCGTGGATGACAGTGG |
| SRSF1-Forward | CCGCAGGGAACAACGATTG |
| SRSF1-Reverse | GCCGTATTTGTAGAACACGTCCT |
| hsa_circ_0016956-Forward | CATTTTAAGACATCCGTCTTGCTA |
| hsa_circ_0016956-Reverse | GCTTCACTGTCATCTTTTTCCAC |
| GAPDH-Forward | GAACGGGAAGCTCACTGG |
| GAPDH-Reverse | GCCTGCTTCACCACCTTCT |
| PCNXL2-Forward | CCATCCCATAGCACCAGTGTC |
| PCNXL2-Reverse | GGTATTACTGACTTGGTCGTGG |
| U6-Forward | CTCGCTTCGGCAGCACA |
| U6-Reverse | AACGCTTCACGAATTTGCGT |
| GAPDH(divergent)-Forward | TGTACCATCAATAAAGTACCCTGTG |
| GAPDH(divergent)-Reverse | AAATCCGTTGACTCCGACCT |
| si-hsa_circ_0016956-1 | AUUUAGCAAGACGGAUGUCTT |
| si-hsa_circ_0016956-2 | UUAUUUAGCAAGACGGAUGTT |
| si-hsa_circ_0016956-3 | UUAGCAAGACGGAUGUCUUTT |
| si-SRSF1 | GAAGTTGGCAGGATTTAAATT |
| miR-766-3p mimics | ACUCCAGCCCCACAGCCUCAGC |
| miR-766-3p inhibitor | GCUGAGGCUGUGGGGCUGGAGU |
| Biotin labeled circPCNXL2 probe | GACATCCGTCTTGCTAAATA- /3bio/ |
| Biotin labeled circPCNXL2 probe NC | CACATCAGATGTTAATCGCT- /3bio/ |

**Table S3 Primers, siRNAs and probes used in this study..**
