# Supplementary material for: CircPCNXL2 promotes tumor growth and metastasis by interacting with STRAP to regulate ERK signaling in intrahepatic cholangiocarcinoma
Source: Mol Cancer. 2024 Feb 17;23:35. doi: 10.1186/s12943-024-01950-y (PMC10873941; doi:10.1186/s12943-024-01950-y)
Supplement: Supplementary file 12 — Supplementary Material 12 [file 12943_2024_1950_MOESM12_ESM.docx]

**CircPCNXL2 promotes tumor growth and metastasis by interacting with STRAP to regulate ERK signaling in intrahepatic cholangiocarcinoma**

Shuochen Liu^1, #^, Yirui Wang^1, #^, Tianlin Wang^1, #^, Kuangheng Shi^1, #^, Shilong Fan^1^, Chang Li^1^, Ruixiang Chen^1^, Jifei Wang^1^, Wangjie Jiang^1^, Yaodong Zhang^1^, Yananlan Chen^1^, Xiao Xu^1^, Yue Yu^1^, Changxian Li^1, *^, Xiangcheng Li^1,2, *^.

**Materials and methods**

**Fluorescence in situ hybridization (FISH) assay**

FISH Probe Mix for circPCNXL2 was synthesized by RioBio (China) and FISH assays were performed using Fluorescent In Situ Hybridization Kit (RioBio, China). Cells were fixated with 4% paraformaldehyde and then incubated with probe overnight at 37 °C. Nuclei were stained with DAPI and photographed by THUNDER Imaging Systems (Leica, Germany).

**Actinomycin D assay**

HuCCT1 or RBE cells were seeded in a 6-well plate at a density of 2× 10^5^ cells/well overnight and treated with 2 µg/ml actinomycin D (MCE, USA) for 4, 8, 12, and 24 h. The expression of circPCNXL2 and PCNXL2 mRNA was analyzed using qRT–PCR.

**RNase R treatment**

The extracted RNA from HuCCT1 and RBE cells was divided into two groups. In the RNase R group, 1 µg of RNA was treated with 2 U of RNase R (Geneseed, China), while in the control group, 1 µg of RNA was mixed with RNase-free water without RNase treatment. Then, qRT-PCR was employed to analyze the expression of circPCNXL2 and PCNXL2 mRNA.

**Nuclear and cytoplasmic extraction**

Nuclear and cytoplasmic fractions were isolated using the reagents in a PARIS™ kit (Thermo, USA). In brief, HuCCT1 and RBE cells were lysed in Cell Fraction Buffer on ice for 10 min. Subsequently, after centrifugation at 500 g for 3 min at 4 °C, the supernatant was collected as the cytoplasmic fraction. Then, the pelleted nuclei were washed with Cell Fraction Buffer and used as the nuclear fraction.

**RNA pull‑down assay**

The biotin-labeled circPCNXL2 probe and control probe (RioBio, China) were incubated with streptavidin magnetic beads (RioBio, China) at room temperature for 30 min to generate probe-coated beads. Lysates from HuCCT1 and RBE cells were saved as a 100ul input group, and the rest of the lysates were incubated with probe-coated beads at 4 °C overnight. Then, the pulled down miRNA was extracted and analyzed by qRT–PCR. The pulled down proteins underwent analysis through silver staining, mass spectrometry and western blotting.

**RNA immunoprecipitation (RIP) assay**

RIP assays were conducted using the RNA Immunoprecipitation Kit (Geneseed, China) following the provided instructions. Anti-IgG, anti-AGO2 or anti-STRAP antibodies were incubated with magnetic beads at 4°C for 2 h. Then, the antibody-bead complexes were incubated with cell lysates from HuCCT1 and RBE cells at 4 °C overnight. The bound RNAs were eluted and reverse transcribed to cDNA and subsequently detected by qRT–PCR.

**Co-Immunoprecipitation assay**

Cells were lysed with NP-40 Substitute (Beyotime, China). Protein complexes were captured using anti-STRAP or anti-MEK1/2 antibodies and IgG at 4 °C overnight and protein A+G magnetic beads were used to capture the antigen antibody complex the next day. After being mixed at room temperature for 2 h, the supernatant was discarded and obtained the antigen antibody complex for subsequent experiments. The protein complexes were separated by SDS-PAGE and detected with corresponding antibodies by western blot assays.

**Dual-luciferase reporter assay**

Wild-type and mutant circPCNXL2 and SRSF1 fragments were constructed and inserted downstream of the luciferase reporter gene in the reporter plasmid pRL-SV40 (GenePharma, China). 293T cells were seeded in 24-well plates and cultured to reach 30% confluence within 24 h. Subsequently, the reporter plasmids were transfected into the cells using Lipofectamine 2000. Cells were co-transfected with different combinations of plasmids harboring the 3′-untranslated region (3′-UTR) of assayed genes and miRNA mimics or the negative control. After 48h, the activities of both firefly luciferase and Renilla luciferase were measured with a Dual-Luciferase Reporter System Kit (Vazyme, China).

**Real-time quantitative PCR (qRT-PCR)**

The total RNA from tissues or cells was extracted using RNA-Quick Purification Kit (Yishan, China). HiScript III 1st Strand cDNA Synthesis Kit (Vazyme, China) was used to reverse transcribe extracted RNA into cDNA. Subsequently, qRT-PCR was conducted with AceQ qPCR SYBR Green Master Mix (Vazyme, China) on the 7900HT Fast Real-Time PCR System (Applied Biosystems, MA, USA). The relative expression was normalized to GAPDH or U6. The primer sequences are listed in Table S3.

**Cell culture and transfection**

Human intrahepatic biliary epithelial cell line (HiBEC), human embryonic kidney 293T (293T), human cholangiocarcinoma cell line RBE, HCCC-9810, HuCCT1 and QBC939 were purchased from the Cell Bank of Chinese Academy of Sciences (Shanghai, China). All the cells were cultured in DMEM High Glucose (KeyGEN BioTECH, China) containing 10% fetal bovine serum (Gibco, USA) and 1% penicillin-streptomycin solution (NCM, USA) at 37°C with 5% CO2. Small interfering RNAs (siRNAs), miR-766-3p mimics or inhibitors were synthesized by GenePharma (Shanghai, China). Lipofectamine TM 2000 (Invitrogen, US) was used for transfection following the manufacturer’s protocol. The sequences of siRNAs, mimics and inhibitors are listed in Table S1. For stable transfected cell lines, cells were transfected using lentiviruses which express negative control and si-circPCNXL2-3. ShRNA was transfected with polybrene (5mg/ml, Sigma) at 10 MOI (multiplicity of infection). Stable cell lines were selected by treating with puromycin (10 μg/mL) for 3 consecutive days after 72 hours of transfection.

**Western blotting**

Tissue or cell samples were lysed by RIPA Lysis Buffer (Beyotime, China) with PMSF. We performed Western blot according to the manufacturer’s protocol. Ultra-sensitive ECL chemiluminescent substrate (Biosharp, China) was applied to detect the level of protein. The antibodies are listed in Table S4.

**Immunohistochemistry (IHC)**

The xenograft tissues were deparaffinized and rehydrated using alcohol before antigen retrieval with citrate antigen retrieval solution (Beyotime, China). Next, the sections were blocked with 5% normal goat serum containing 0.1% Triton X-100 and 3% H_2_O_2_ in PBS for 1h at room temperature. Then, the sections were incubated with specific primary antibodies at 4℃ overnight and then incubated with secondary antibody. Finally, IHC staining was detected using Light microscopy (Nikon, Japan).

**CCK-8 assay**

The CCK-8 assay kit (Dojindo, Japan) was employed to assess cell proliferation. Cells were seeded at a density of 1×10^3^ cells/well in a 96-well plate the night before. Subsequently, 100μl medium containing 10μl CCK8 reagent was added into each well daily. After 2h incubation, the absorbance at 450nm was measured by Multi-Mode Microplate Reader (Biotek, USA).

**Edu assay**

EdU cell proliferation kit (Beyotime, China) was used for EdU assay to assess cell proliferation. Cells were planted in 24-well plates at 80% confluence, cultured for 24h, and then treated with 10μM EdU in medium for 2h. The cells were fixed in 4% paraformaldehyde and permeabilized with 0.3% Triton in PBS for 15 min. Sequentially the cells were stained with Alexa Fluor 555 azide for 30 min and DAPI for 10 min in the dark. The images were captured under a fluorescence microscope (Leica, Germany).

**Clone formation assay**

Cells were seeded in 6-well plates at a density of 1×10^3^/well and incubated for 10 days. The cells were fixed and then stained with Crystal Violet Staining Solution (Beyotime, China) for 30 min. The colonies were photographed and the number of it was counted by Image J software.

**Transwell assay**

Transwell assays were performed to assess migration. 5×10^4^ cells were seeded into Transwell BD Matrigel (Corning, USA), with serum-free medium in the upper layer and medium containing 20% fetal bovine serum in the lower layer. After 2 days incubation, migrating cells were fixed with 4% paraformaldehyde and then stained with Crystal Violet Staining Solution (Beyotime, China). The images were shot under a light microscope (Olympus, Japan). The number of migration cells was measured by Image J software.

**Wound healing assay**

Cells were plated in 6-well plates at a density of 5× 10^5^ cells per well. When the cell density reached 90%-100%, a straight wound was created using a 200-µl pipette tip. Wound images were captured using light microscopy (Olympus, Japan). The proportion of area healed was measured by Image J software.

**Supplement Figure 1 Identification of circPCNXL2 and circPCNXL2 is upregulated in ICC. (a)** The level of circPCNXL2 is upregulated in ICC tissues compared with normal tissues in GSE181523 datasets. **(b)** The ROC curve analysis of circPCNXL2 in 76 ICC patients.

**
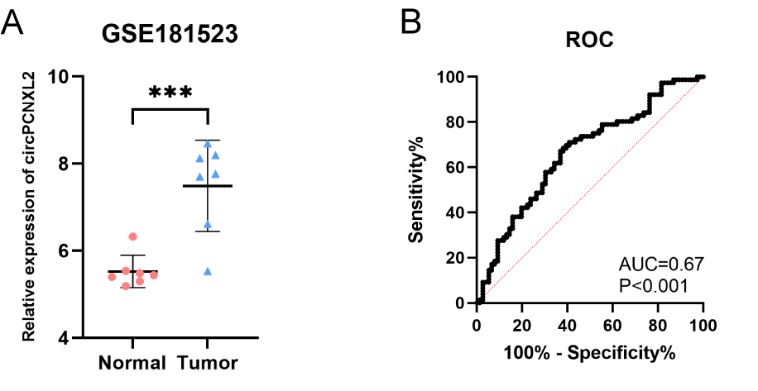
**

**Supplement Figure 2 The efficiency of CircPCNXL2 knockdown or overexpression in vivo. (a-b)** The expression of circPCNXL2 in xenografts was measured by qRT-PCR.

**
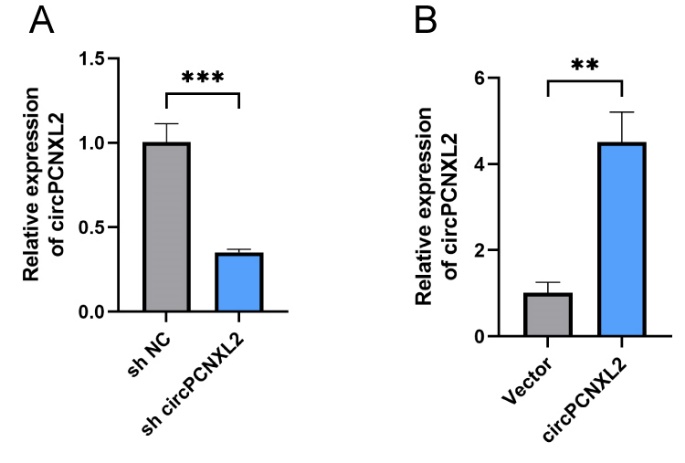
**

**Supplement Figure 3 CircPCNXL2 promotes the activation of MEK/ERK pathway in ICC in vitro. (a)** GO enrichment analysis of differentially expressed genes. **(b)** CCK8 assay was conducted to evaluate the proliferation in RBE cells, scale bar = 50μm. **(c)** EdU assay of RBE cells. **(d)** Colony formation assay of RBE cells. **(e)** Transwell assay was used to evaluate the migration in RBE cells, scale bar = 100μm. **(f)** Wound healing assay of RBE cells, scale bar = 100μm. *P<0.05; **P<0.01; ***P<0.001**
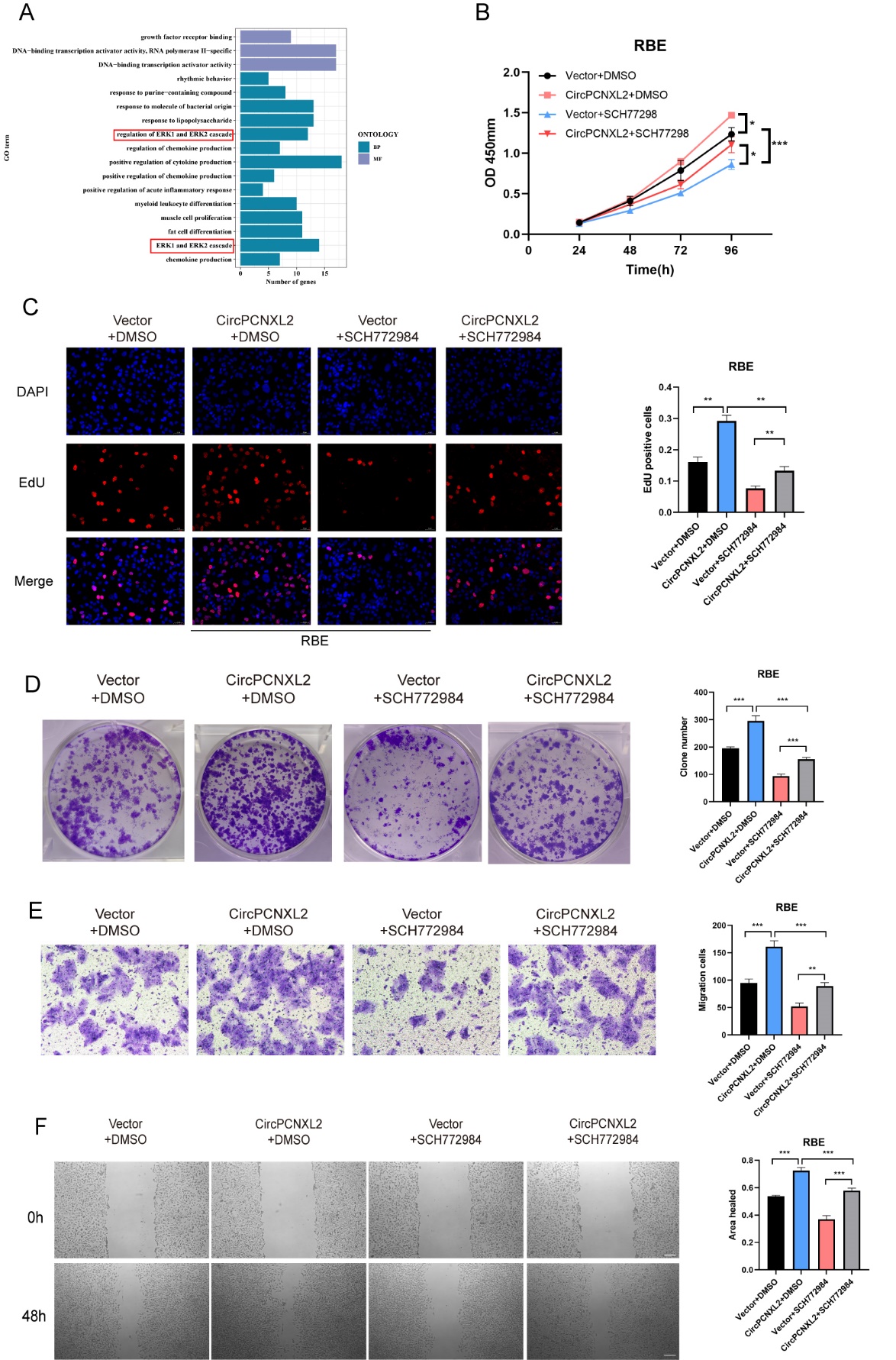
**

**Supplement Figure 4 CircPCNXL2 acts a miR-766-3p sponge and promote SRSF1 expression. (a)** The enrichment of 4 putative miRNAs was evaluated in the RNA pull down by circPCNXL2 probe in RBE cells. **(b)** The enrichment of circPCNXL2 and miR-766-3p was confirmed by RIP with AGO2 or IgG antibody in RBE cells. **(c-d)** The efficiency of miR-766-3p mimics or inhibitor was certified by qRT-PCR. **(e)** The functions of miR-766-3p on the proliferation of RBE cells was detected by colony formation assay. **(f)** The functions of miR-766-3p on the migration of RBE cells were detected by transwell assay, scale bar = 100μm. **(g-h)** The mRNA and protein levels of SRSF1 were evaluated in RBE transfected with miR-766-3p mimics. **(i)** The expression of SRSF1 in ICC tissues from TCGA dataset. **(j-k)** The efficiency of SRSF1 siRNA or plasmid was certified by qRT-PCR. **(l)** The functions of SRSF1 on the proliferation of RBE cells were detected by colony formation assay. **(m)** The functions of SRSF1 on the migration of RBE cells were detected by transwell assay, scale bar = 100μm. **(n)** The expression of miR-766-3p in xenografts was measured by qRT-PCR. **(o-p)** IHC staining of SRSF1 in xenografts, scale bar = 50μm.*P<0.05; **P<0.01; ***P<0.001

**
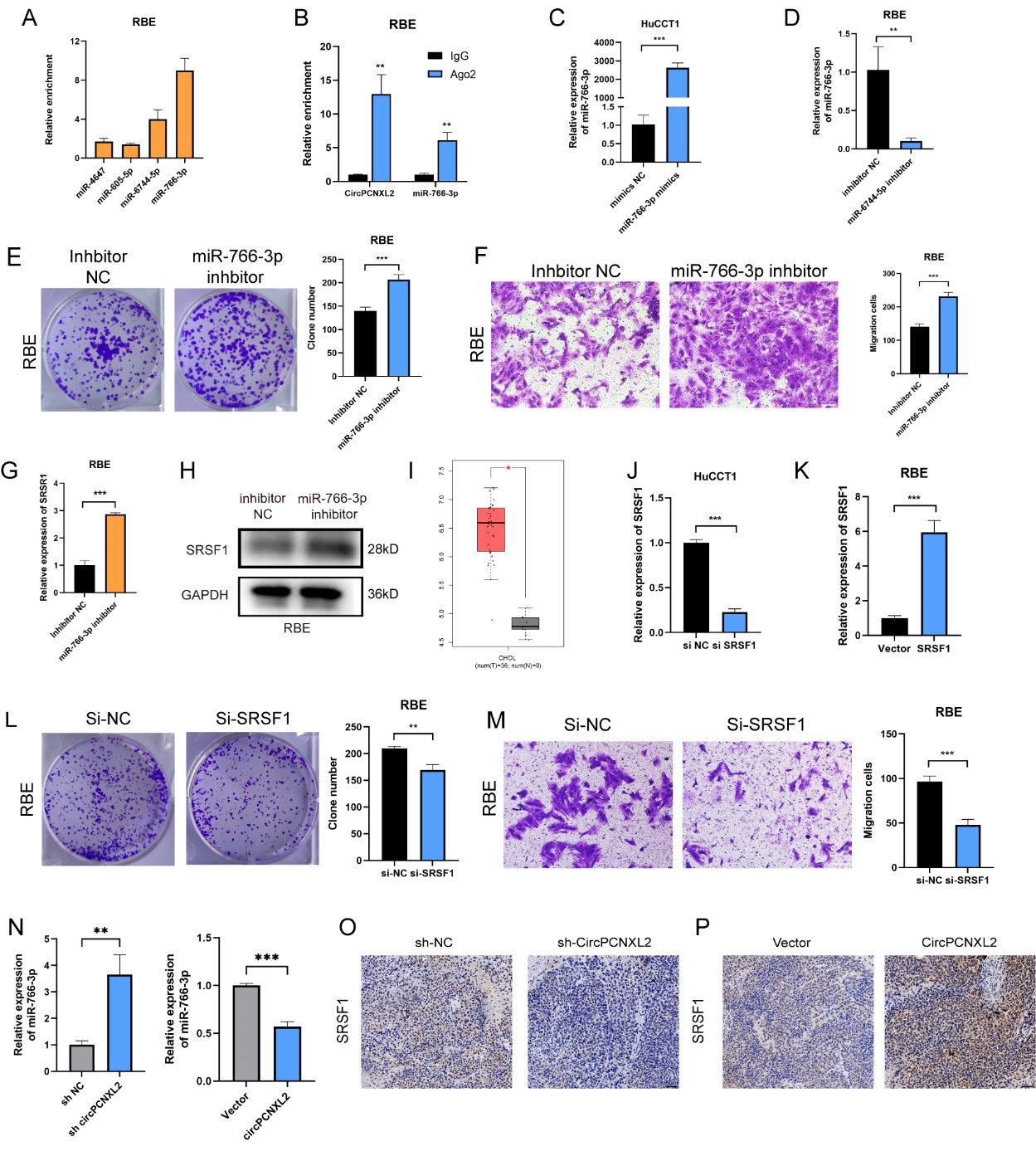
**
